# A comprehensive, large-scale analysis of “terroir” cheese and milk microbiota reveals profiles strongly shaped by both geographical and human factors

**DOI:** 10.1093/ismeco/ycae095

**Published:** 2024-07-11

**Authors:** Françoise Irlinger, Mahendra Mariadassou, Eric Dugat-Bony, Olivier Rué, Cécile Neuvéglise, Pierre Renault, Etienne Rifa, Sébastien Theil, Valentin Loux, Corinne Cruaud, Frederick Gavory, Valérie Barbe, Ronan Lasbleiz, Frédéric Gaucheron, Céline Spelle, Céline Delbès

**Affiliations:** Université Paris Saclay, INRAE, AgroParisTech, UMR SayFood, 91120 Palaiseau, France; Université Paris-Saclay, INRAE, MaIAGE, 78350, Jouy-en-Josas, France; Université Paris Saclay, INRAE, AgroParisTech, UMR SayFood, 91120 Palaiseau, France; Université Paris-Saclay, INRAE, MaIAGE, 78350, Jouy-en-Josas, France; UMR SPO, Université Montpellier, INRAE, Institut Agro, Montpellier, France; Université Paris-Saclay, INRAE, AgroParisTech, Micalis Institute, 78350 Jouy-en-Josas, France; Université Clermont Auvergne, INRAE, VetAgro Sup, UMR 545 Fromage, Aurillac, France; Université Clermont Auvergne, INRAE, VetAgro Sup, UMR 545 Fromage, Aurillac, France; Université Paris-Saclay, INRAE, MaIAGE, 78350, Jouy-en-Josas, France; Genoscope, Institut François Jacob, Commissariat à l’Energie Atomique (CEA), Université Paris-Saclay, 91057 Evry, France; Genoscope, Institut François Jacob, Commissariat à l’Energie Atomique (CEA), Université Paris-Saclay, 91057 Evry, France; Genoscope, Institut François Jacob, Commissariat à l’Energie Atomique (CEA), Université Paris-Saclay, 91057 Evry, France; CNAOL, Maison du Lait, 75009 Paris, France; CNIEL, Maison du Lait, 75009 Paris, France; CNAOL, Maison du Lait, 75009 Paris, France; Université Clermont Auvergne, INRAE, VetAgro Sup, UMR 545 Fromage, Aurillac, France

**Keywords:** cheese, milk, core microbiome, ecological drivers, signatures, terroir

## Abstract

An exhaustive analysis was performed on more than 2000 microbiotas from French Protected Designation of Origin (PDO) cheeses, covering most cheese families produced throughout the world. Thanks to a complete and accurate set of associated metadata, we have carried out a deep analysis of the ecological drivers of microbial communities in milk and “terroir” cheeses. We show that bacterial and fungal microbiota from milk differed significantly across dairy species while sharing a core microbiome consisting of four microbial species. By contrast, no microbial species were detected in all ripened cheese samples. Our network analysis suggested that the cheese microbiota was organized into independent network modules. These network modules comprised mainly species with an overall relative abundance lower than 1%, showing that the most abundant species were not those with the most interactions. Species assemblages differed depending on human drivers, dairy species, and geographical area, thus demonstrating the contribution of regional know-how to shaping the cheese microbiota. Finally, an extensive analysis at the milk-to-cheese batch level showed that a high proportion of cheese taxa were derived from milk under the influence of the dairy species and protected designation of origin.

## Introduction

A wide variety of ripened cheeses characterized by distinct organoleptic properties have been produced around the world for decades under contrasting environmental conditions and applying local know-how. The extensive literature on abiotic drivers (salt, temperature, relative humidity, and pH) collected throughout the long history of cheese production and on the multi-omic characterization of a large panel of cheeses has provided an overview of the cheese maturation process and the complexity of cheese microbial communities, and a clearer understanding of their metabolic activities [1–4]. Ripened cheeses are thus a particularly relevant model to test their link with the concept of “terroir,” which implies that local ecological drivers, such as climate, soil, rainfall, and human activities, determine the phenotype of raw materials and the operational adjustments of processing, and may thereby alter the assembly of food fermentation microbiota. Originally coined to describe the link between local growing conditions and the organoleptic characteristics of wine [5], the concept of “terroir” has since been extended to several other fermented foods [6–10]. Nevertheless, the existence of a microbial “terroir” among cheeses remains a controversial issue. The scientific community struggles to identify any possible correlations between the diversity and composition of milk and cheese microbiota and the geographic scope and technological characteristics that define a cheese type. Two extensive studies on cheeses from 10 and 16 different countries on two continents (Europe and the United States, respectively) reached opposite conclusions regarding the relationships between the geographical origin of cheeses and microbial biodiversity [4, 11]. However, the taxonomic level of analysis in both studies was limited to the genus level, and a coarse-grained typology of cheeses was applied. These limitations did not enable a detailed analysis of the relationships between geographic origin, production process, and microbiota diversity. By contrast, using species-level data on hard or semi-hard cheeses from Ireland, Cyprus, or Switzerland, Kamilari *et al.* [12] and De Respinis *et al.* [13] showed that geographical region and the environment created by cheesemakers during the production and ripening processes are factors that structure the cheese microbiota. Few studies, including none of the above, have considered other compartments of the food chain, such as milk, grassland, phyllosphere, or soil, in parallel with the cheese microbiota [14, 15] and identified possible microbial transfers between them.

Cheeses labeled as having a Protected Designation of Origin (PDO), a system strictly regulated by the European Union (EU), are representative of foodstuffs produced, processed, and prepared in a defined geographical area, according to recognized traditional processes. They represent a valuable object of study to assess the involvement of local ecological factors in structuring the microbial community. Thanks to the involvement of PDO cheese stakeholders and more than 200 farmers, we therefore sampled 2702 rinds and cores of 44 French PDO cheeses and their associated milks spread over an area of more than 600 000 km^2^ and collected detailed data on their production conditions in 2017. This sampling covers a panel of seven cheese families that have similarities with the most ripened cheeses produced throughout the world. We characterized the bacterial and fungal communities of the milk and cheeses using amplicon-based sequencing (16S rRNA and ITS2) and association network analyses to identify systemic taxa. The dataset generated on the individual milk-cheese continuum is unprecedented in terms of its comprehensiveness, methodological consistency, and the completeness and accuracy of the associated metadata. We have thus been able to carry out an original, sound, and generalized analysis of the microbial, geographical, and human drivers of the assembly and richness of microbial communities in milk and “terroir” cheeses.

## Materials and methods

### Study design and sample collection

All cheese and milk samples from 386 batches across the 44 PDO areas were sampled individually in 2017 by the PDO cheese stakeholders themselves at each location using the same methodology set up by the project committee composed of researchers and CNAOL representatives (Supplementary Table S1). Each PDO sampled an average of 8.7 batches (min. 1, max. 13). All samples were anonymized and coded randomly from PDO1 to PDO44. For each cheese batch, the corresponding milk was sampled beforehand from the cheese-making vat before adding any starter culture. Three hundred seventy milk samples (180 ml) were taken in sterile plastic containers, kept cold (4°C), and then frozen (−20°C) within 4 h of collection, while cheese samples were maintained at 4°C until the analyses were performed. Three individual cheeses from the same production batch, chosen at random, were tested to account for production variation. They were collected at the end of the ripening period, as defined in the specifications for each PDO, i.e. when they were ready to be brought to market and eaten. A total of 2316 subsamples of rinds and cores from 1158 cheeses were collected by scraping the surface of the rind with a sterile razor blade and cutting a piece from the inside of the cheese, respectively, then homogenized with a sterile mortar and pestle and transferred into sterile tubes of 50 g.

These cheese samples were used for cell counts and pH measurement, as described in [16].

### DNA extraction, PCR amplification, and massive sequencing

DNA was extracted from a 250-mg aliquot of cheese sub-samples using a lysis step and a phenol/chloroform method and purified with the Genomic DNA Clean & Concentrator-10 kit (Zymo Research). Two primer pairs were used to amplify two ribosomal RNA (rRNA) barcode loci: a 16S rRNA gene fragment targeting the V3-V4 regions to characterize bacterial diversity was amplified using the primers 16S_V3F according to [17] and 16S_V4R [18], while a fungal gene fragment targeting the ITS2 region to characterize fungal diversity was amplified using the primers ITS3f according to [19] and ITS4_KYO1 [20]. Two thousand six hundred ninety-eight milk and cheese samples were successfully amplified from the DNA samples. Sequencing was then carried out on the Illumina HiSeq 2500 platform (2 × 250) by Genoscope (Evry, France). Detailed methods describing DNA extraction, amplification of 16S rRNA and ITS2 target regions, and amplicon sequencing are provided in Supplementary Methods.

### Quality control of the sequences and bioinformatics analyses

The resulting sequences were analyzed using a workflow combining dada2 v.1.16 [21] and FROGS 3.2.2 software [22]. Briefly, after dereplication, error corrections, and chimera removal, amplicon sequence variants (ASVs) were affiliated with the FROGS *affiliation_OTU* tool with silva v.132 [23] and DAIRYDB v1.1.2 [24] databanks for 16S data, and with UNITE v7.2 and a collection of personal sequences. Finally, all ASVs with a specificity value [25] in the negative controls >0.7 were removed. Detailed methods describing the bioinformatic processing of ITS and 16S amplicon sequences are provided in Supplementary Methods.

### Statistical methods

Statistical analyses were conducted using R (v.4.3.1), phyloseq (v.1.34) [26], and PLN models (v.1.0.1) [27]. Data were rarefied for alpha and beta diversity analyses but not for network analyses. Alpha-diversity analyses were performed for each factor using observed ASV richness and a one-way ANOVA. Beta diversity analyses were performed using Bray–Curtis distances and nonmetric multidimensional scaling (NMDS), and the effect of each factor was assessed using PERMANOVA.

For microbial transfer indicator determination, production batches were filtered to keep only complete batches (milk and cheese samples) and rarefied before computing the number of shared ASVs between milk and core/rind and their respective abundance in each compartment. A logistic regression model with no interactions was used to estimate the effect of technological family, production type, and dairy species on the probability of being shared.

For network-based analysis, rind and core samples were aggregated at the production batch level and used to reconstruct a synthetic variable capturing the main structuring factors. Hierarchical clustering (ward linkage) on the Bray–Curtis distances averaged over 16S and ITS2 markers identified five clusters, each representing a different synthetic ecological niche (see Supplementary Fig. S1). ASV was then aggregated at the species level, and a high prevalence of 20% in one cluster or >10% in the whole dataset) was selected for network reconstruction.

Network reconstruction was performed on raw abundance tables, with differences in sequencing depths corrected using the GMPR normalization method [28]. A network with nodes corresponding to 75 bacterial and 57 fungal species was computed using the PLN network method based on a graphical lasso. Finally, microbial modules of species with similar connectivity patterns were identified using stochastic-block models.

## Results

### Microbial composition of French PDO cheeses and milk

**Figure 1:**
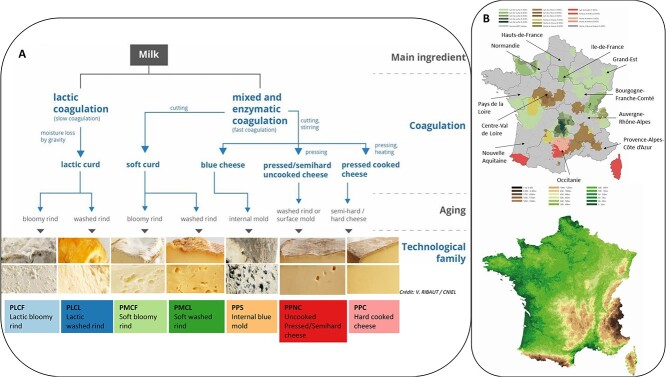
Characteristics of cheese families and geographic distribution of sampling locations. (A) classification scheme shows the different cheese families in terms of fundamental technological characteristics according to [29] but with additional representative cheese (core and rind) pictures. (B) Two maps of France, including (i) the PDO areas (cow’s milk, goat’s milk, sheep’s milk, both or all three) and the delimitation of the 10 French administrative regions and (ii) the different reliefs and mountain massifs (plateau, hill, plain, and mountain).

A total of 2702 cheese and milk samples were obtained from 386 farmhouse or corporate dairy PDO producers across France (Supplementary Fig. S2). Cheese samples represented 44 PDOs and were assigned to seven cheese families [29] (Fig. 1A and Supplementary Results). Within each PDO, cheeses were selected to cover the diversity of production practices within the agricultural and technological specifications of each PDO (Fig. 1 and Supplementary Table S1).

All samples were successfully sequenced and passed sequence quality filters for bacterial and fungal communities. After sequence pre-processing (Supplementary Results and Supplementary Table S2), the retained ASVs were assigned to 1702 bacterial species (1215 for milk, 810 for cheese) spanning 661 genera (543 for milk, 285 for cheese), and 1156 fungal species (1097 for milk, 273 for cheese) spanning 544 genera (528 for milk, 136 for cheese). By retaining only those ASVs whose relative abundance was higher than 0.1% and which were detected in triplicate in our dataset, we were able to count 2235 bacterial ASVs and 1630 fungal ASVs from milk samples, 1304 bacterial ASVs, and 254 fungal ASVs from cheese samples. Of these, 50% of bacterial ASVs and 3% of fungal ASVs from milk and 22% of bacterial ASVs and 10% of fungal ASVs from cheese could only be identified down to the genus level and were not assigned to any known species.

At a global level, the taxonomic assignment of ASVs from milk and cheeses revealed their affiliation to three main bacterial phyla (*Actinomycetota*, *Bacillota*, and *Pseudomonadota*) and two main fungal phyla (*Ascomycota* and *Mucoromycota*) for cheeses, and to three main fungal phyla for milk (*Ascomycota*, *Basidomycota*, and *Mucoromycota*). Their relative abundance varied according to localization (milk, cheese rind, and core) and technological family (see Supplementary Fig. S3A and B for the detailed distribution of the dominant fungal and bacterial species in milk and cheese samples).

The 10 fungal species that were the most abundant across the full dataset represented more than 75% of the total fungal population in most individual cheese samples. They included well-known species commonly added as ripening cultures in cheese-making (*Geotrichum candidum* (teleomorph *Galactomyces candidus*), *Debaryomyces hansenii*, *Diutina catenulata*, *Fusarium domesticum*, *Kluyveromyces lactis*, *Mucor lanceolatus*, *Penicillium camemberti*, *Penicillium roqueforti*, and *Scopulariopsis flava*). However, their relative abundances differed significantly across sampling localization (core or rind) and technological families: PLCF (lactic bloomy rind) and PMCF (soft bloomy rind) generally displayed higher relative abundances of *G. candidum* and *P. camemberti*, whereas *P. roqueforti* was more abundant in the PPS (internal blue mold) population. Similarly, the 10 most dominant bacterial species were well-documented cheese colonizers (*Brevibacterium aurantiacum*, *Corynebacterium casei*, *Corynebacterium variabile*, *Lacticaseibacillus casei*, *Lactobacillus delbrueckii*, *Lactococcus lactis*, *Leuconostoc mesenteroides*, *Leuconostoc pseudomesenteroides*, *Psychrobacter aquimaris*, *Staphylococcus equorum*, and *Streptococcus thermophilus*). However, on average, these species constituted <50% of the bacterial community of the cheese rind and <75% that of the cheese core, except for PLCF (lactic bloomy rind) cheeses, where more than 80% of the sequences belonged to *L. lactis* and *L. mesenteroides*.

Taxonomic assessments of the microbial ASVs revealed that bacterial and fungal populations in the milk were highly diverse and variable, with <40% of the community accounted for by the 12 most dominant fungal and bacterial species present.

### Microbial richness varies by dairy animal species and technology

**Figure 2:**
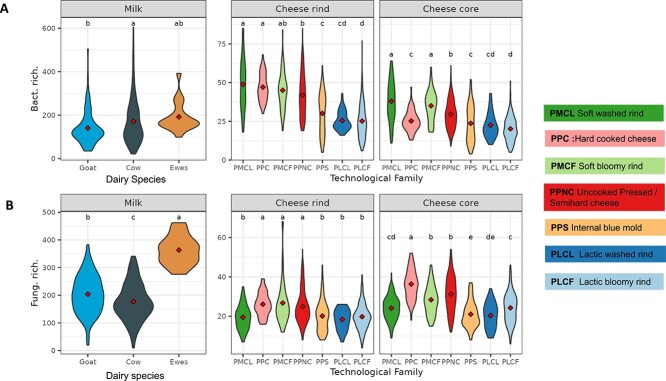
α-Diversity analyses based on microbial richness (observed number of ASVs) in milk microbiota (*n* = 370), in cheese rind microbiota (*n* = 1148) and in cheese core microbiota (*n* = 1148) violin plots show the distribution of observed richness after rarefaction for bacterial (A) and fungal (B) ASVs grouped by localization (milks, cheese cores, and cheese surfaces) and according to animal species for the milks and to technological family for the cheeses. All α-diversity values were calculated based respectively on rarefaction to 2495 bacterial reads and 9565 fungal reads per milk sample and 13 827 bacterial reads and 8187 fungal reads per cheese sample to account for differences in sequencing depth between samples. The significance of differences between samples (ANOVA followed by posthoc Tukey’s HSD test) is marked by lowercase letters so that samples that share at least one letter are not significantly different, but samples that do not share a letter are significantly different.

A variability in ASV richness was observed as a function of technological families (16S rRNA gene markers, 139–867 ASVs; ITS2 markers, 194–1521 ASVs; Fig. 2A and B). Samples of cheese rinds assigned to the PMCF (soft bloomy rind), PPC (hard cooked cheese), and PPNC (uncooked pressed/semihard cheese) cheese families had a significantly higher fungal richness (average of 26 ASVs) than the PLCF (lactic bloomy rind), PLCL (lactic washed rind), PMCL (soft washed rind), and PPS (internal blue mold) families (average of 19 ASVs). For the bacterial community, cheese rinds from PMCF (soft bloomy rind), PMCL (soft washed rind), PPC (hard cooked cheese) (average 47 ASVs), and PPNC (uncooked pressed/semihard cheese) (42 ASVs) had significantly higher richness than PLCF (lactic bloomy rind) and PLCL (lactic washed rind) families (26 ASVs). Cheese cores from PMCL (soft washed rind) and PMCF (soft bloomy rind) technological families had a significantly higher bacterial richness (average of 36 ASVs) than PLCF (lactic bloomy rind), PLCL (lactic washed rind), and PPS (internal blue mold) families (average of 22 ASVs). In contrast, cheese cores from PPC (hard cooked cheese) had a significantly higher fungal richness (average of 36 ASVs) than PLCF (lactic bloomy rind), PLCL (lactic washed rind), PPS (internal blue mold), and PMCL (soft washed rind) families (average of 22 ASVs).

The balance between the prokaryotic and fungal richness in each milk and cheese sample was explored. The microbiota from milk and cheese cores displayed a variable proportion of bacterial and fungal taxa. However, richness values were distributed close to the 1:1 line, indicating that communities were equally diverse in both microbial domains (Supplementary Fig. S4A). However, differences in the proportions of prokaryotic and fungal richness were observed between cheese technological families (Supplementary Fig. S4B). For instance, cheese cores ranged from having richer fungal diversity (in the PPC (hard cooked cheese) technology family) to having richer prokaryotic diversity (in the PMCL (soft washed rind) technology family). Unlike the milk and cheese cores, cheese rinds appeared to have richer prokaryotic than fungal diversity regardless of the technology family (Supplementary Fig. S4A and B).

In summary, these results revealed greater prokaryotic richness in cheese rinds and a similar contribution of the prokaryotic and fungal domains to the richness of the microbiota of milk and cheese cores, except for two technological families (PMCL (soft washed rind) and PPC (hard cooked cheese)), whose richness appeared to be higher for prokaryotes and fungi, respectively.

### Milk has a core microbiota, but not cheeses from France

We examined the species widely detected at high levels of relative abundance (Supplementary Fig. S5) and defined the core microbiome of milk and cheeses as that with a relative abundance >0.1% in 90%–100% of the samples. Despite differences in the milk microbial communities that depended on the dairy animal species, a core milk microbiome could be detected and included four microbial species (*Moraxella osloensis*, *L. lactis*, *Romboutsia timonensis*, and *Geotrichum candidum*) (Supplementary Fig. S5A, Supplementary Table S3). These microbial core species represented a minority of the microbial species (1/1367, 0.37% for fungi, and 3/1230, 0.24% for bacteria). However, they accounted for an average of 12.1% and 16.2% of all fungal and bacterial reads, respectively.

Using the same rules of high relative abundance (>0.1%) and ubiquity (90%–100% of the cheese samples), we found no core microbial species in the cheese community (rind and core) (Supplementary Fig. S5B and C, Supplementary Table S3).

Applying the same rules within each cheese technological family, we found seven fungal and thirteen bacterial species forming a core in at least one technological family. The three fungal species, *Geotrichum candidum*, *Scopulariopsis flava*, and *D. hansenii*, were the most abundant and prevalent in PPC (hard cooked cheese), accounting for 39%, 6%, and 20% of cheese cores and 0%, 22%, and 24% of reads in cheese rinds. At the same time, *Penicillium roqueforti* was explicitly detected in PPS (internal blue mold) cheese cores (68%) and rinds (10%). The core bacterial species *L. lactis* was detected in most technological families except for PPC (hard cooked cheese), and PMCL (soft washed rind), and represented 35%–75% of reads in cheese cores and 23%–34% in cheese rinds. The PPC (hard cooked cheese) family displayed the largest bacterial core microbiota with ten species found in all PPC (hard cooked cheese) cheese samples (Supplementary Table S3): *Alkalibacterium gilvum*, *Brevibacterium jeotgali*, *B. aurantiacum*, *Brachybacterium paraconglomeratum*, *S. equorum*, *L. delbrueckii subsp. lactis*, *C. casei*, *Halomonas Group boliviensis alkaliantarctica*, *Corynebacterium nuruki*, and *S. thermophilus*, which together accounted for 69% of all reads in PPC (hard cooked cheese) cheese rinds. *L. mesenteroides* was detected as a core species in both PLCF (lactic bloomy rind) and PMCF (soft bloomy rind) cheese cores with a relative abundance of 13.6% and 3.6%, respectively.

Whatever the technological family, the core microbial species identified in the inner parts of cheeses were well known to be cheese starters (lactic bacteria) or ripening cultures. In cheese rinds, bacterial species not known to be used as starters represented the majority of reads.

### A cheese microbiome highly structured by a few network modules

The potential associations between the relative abundances of microbial taxa in cheeses that remained after correction for shared ecological niches and technological family were investigated using a network analysis that combined bacterial and fungal ASVs.

**Figure 3:**
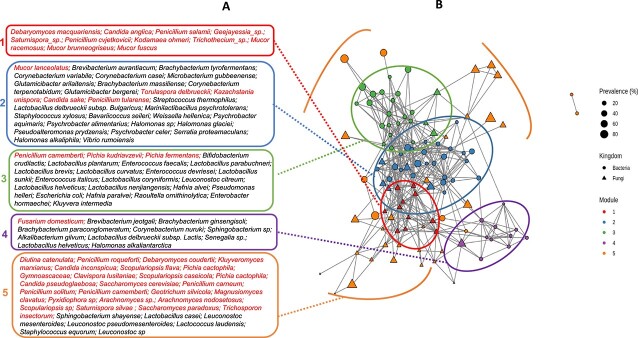
Association network of microbial species from the French PDO cheeses. Nodes are colored according to the modules and their size is proportional to the prevalence of the species in the dataset. Bacterial nodes are represented by circles, and fungal nodes by triangles. (A) Shows the modules and (B) displays the names of the species contained in each module (red for fungi and black for bacteria).

The final network comprised 132 nodes (75 bacterial and 57 fungal species) and 469 edges (Fig. 3), 467 of which were positive and reflecting positive interactions between two species, while two were negative, reflecting negative interactions. The substantial imbalance between positive and negative edges was typical of microbial networks reconstructed from relative abundance data, as exclusions are harder to infer correctly than co-occurrences [30]. Network density was low (0.054), which is usual for microbial networks. Regarding edges, 235 (50%) were found between bacterial nodes, 137 (29%) between fungal nodes, and 97 (21%) between bacterial and fungal nodes. The latter 97 would have been missed by methods focused on a single marker gene (16S or ITS2) at the time.

A cluster analysis revealed that the network was organized into five modules (Fig. 3A), i.e. blocks of interconnected nodes. These modules comprised 11–35 nodes, and their composition in fungal and bacterial nodes was variable (Fig. 3B, Supplementary Table S4). Modules 1–4 exhibited high intra-module connectivity, unlike module 5, which was composed of poorly connected satellite nodes (Supplementary Fig. S6A and B). This type of core-periphery organization has been observed in many other biological networks. Remarkably, module 1, the most closely connected with the other modules, was exclusively composed of fungal nodes with low relative abundance and low prevalence. Module 2 comprised many highly prevalent taxa corresponding to microorganisms that are often deliberately inoculated by cheese makers, such as *S. thermophilus*, *L. delbrueckii subsp. bulgaricus*, *B. aurantiacum*, *Brachybacterium tyrofermentans*, *C. casei*, *Glutamicibacter arilaitensis*, and *Staphylococcus xylosus*, as well as many environmental psychrophilic and halotolerant bacteria, such as *P. aquimaris*, *P. celer*, *Psychrobacter alimentarius*, *Halomonas glaciei*, *H. alkaliphila*, *Marinilactibacillus psychrotolerans*, and *Pseudoalteromonas prydzensis* (Supplementary Table S4). Module 3 contained *Penicillium camemberti*, a fungal aerobic species, and numerous *Bacillota* (mainly lactic acid bacteria) and *Enterobacteriaceae*, facultative anaerobic bacteria commonly found in the cheese core. Module 4 consisted exclusively of aerobic microbial species detected on the surface of PPC (hard cooked cheese).

Overall, the five modules were not highly connected (Supplementary Fig. S6B), with between-module connectivity of <5% for all modules except for module 1 (connectivity of 12% to module 2 and 11% to module 5). This finding suggested that the microbiota was organized into mostly independent network modules, with species that interacted preferentially with others within the same module, and that Module 4 (species adapted to a unique cheese-making habitat: hard-cooked cheeses) might be particularly relevant. Moreover, most of those species had an overall relative abundance lower than 1%, showing that the most abundant species were not those with the most interactions.

### PDO-dependent factors shape the milk and cheese microbiome

**Figure 4:**
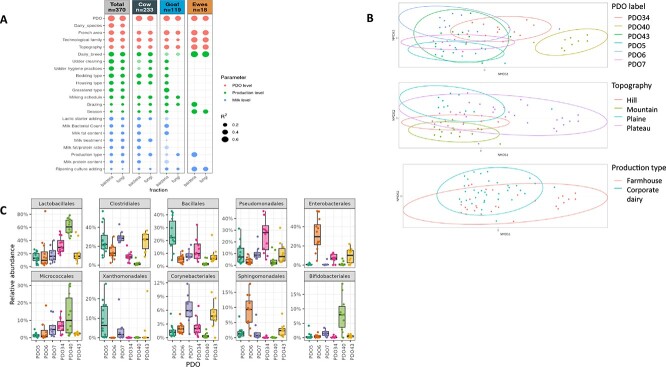
Environmental, farming and technological parameters contributing to milk microbiota shaping. (A) correlations between the beta-diversity based on bray-Curtis dissimilarities of the total milk dataset (*N* = 370), and environmental, farming and technological parameters. The parameters were sorted by category as PDO and PDO-driven variables, farming practices associated to each production and milk sample specific descriptors, and then by R2 value, represented by the size of the label, with a threshold set to R2 > 0.2. Dark colors indicate significant correlations with p values below 0.05. Data are presented in column alternately for bacteria and fungi. (B, C) Main contributors to cow’s milk bacterial community shaping. Focus on the milk from the six PDOs with the lowest intra-PDO dispersion (*N* = 63). (B) Non metric multi-dimentional scaling ordinations based on bray-Curtis dissimilarities. PERMANOVA analyses were performed to test the effects of PDO label (R2 = 0.386, *P*-value < .001), topography (R2 = 0.220, *P*-value < .001), and production type (R2 = 0.054, *P*-value < .001). (C) Box plots showing the relative abundance of the bacterial orders above 10% relative abundance, in the individual samples of cow’s milk across the six PDOs.

Regarding milk, the PDO variable explained most of the variance observed within the dataset (57.8% and 52.7% for richness and 37.3% and 27.6% for beta-diversity for bacteria and fungi, respectively) (Fig. 4A, Supplementary Fig. S7A–C, Supplementary Table S5). The main drivers of the alpha- and beta-diversity of the milk microbiota were PDO-prescribed variables, starting with the dairy species, followed by the French region and the topography (Fig. 4A; Supplementary Fig. S7A–C, Supplementary Table S5). As for farm-specific variables, regarding all dairy species combined, the dairy breed, udder hygiene practices, bedding type, and grassland type appeared to be essential contributors to the observed diversity and composition of milk microbiota (Fig. 4A, Supplementary Fig. S7A–C). Some farming practices differed according to the dairy species, such as the lack of pre-milking udder cleaning in goats (Supplementary Table S1), which may contribute to differences in the microbiota composition of cow’s and goat’s milks. In general, more potent effects were detected on bacterial communities than fungal communities, and with respect to cow’s milk, which in our farm panel was associated with a larger sample size (*n* = 233) and a greater diversity of practices than goat’s and sheep’s milk. The Bray–Curtis dissimilarity matrices were analyzed to identify environmental and technological practices that might contribute to the shaping of the milk microbiota by PDO. Among the total of 28 PDOs produced from cow’s milk, the bacterial microbiota in milk from six PDOs (PDO5, PDO6, PDO7, PDO34, PDO40, and PDO43; *N* = 63) clustered most significantly by PDO and displayed the lowest intra-PDO sample dispersion (Fig. 4B). These samples were distributed over five technological families (PLCF (lactic bloomy rind), PLCL (lactic washed rind), PPC (hard cooked cheese), PPNC (uncooked pressed/semihard cheese), and PPS (internal blue mold)), four topography profiles, and two French regions (Auvergne-Rhône-Alpes, Bourgogne). We showed that topography and production type (farmhouse vs. commingled milk) could contribute to explaining the PDO-related specificities of the milk bacterial profiles, but that these effects could be confounded with those of the milking schedule and of the type of vat used to collect the milk (PERMANOVA test). For instance, PDO40 milks with atypical bacterial profiles were the only milks collected from wooden vats where raw milk is curdled daily (Fig. 4C).

**Figure 5:**
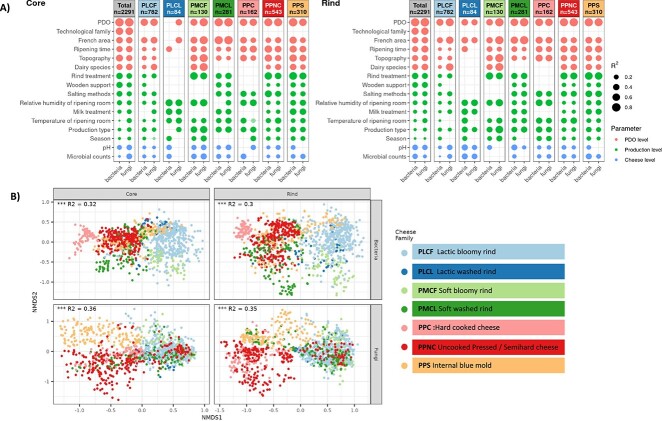
Environmental, farming, and technological parameters contributing to cheese microbiota shaping. (A) Correlations between the beta-diversity based on bray-Curtis dissimilarities of the total cheese dataset (*N* = 2291) and environmental, farming, and technological parameters. The parameters were sorted by category as PDO and PDO-driven variables, practices associated with each production, and cheese sample-specific descriptors, and then by R2 value, represented by the size of the label. Dark colors indicate significant correlations with *P*-values below .05. Data are presented in columns alternately for bacteria and fungi. (B) Microbial community beta-diversity of the total cheese dataset (*N* = 2291) according to the seven cheese families. Non-metric multi-dimensional scaling ordination (NMDS) based on Bray–Curtis dissimilarities. Top: Bacterial communities. Bottom: Fungal communities. Left: Cheese core communities. Right: Cheese rind communities.

The PDO variable explained most of the variance observed within the dataset for all cheese samples combined. In the cheese core, PDO explained 61.9% (resp. 58%) of the variance for richness and 60.2% (resp. 64.7%) of the variance for the beta diversity for bacteria (resp. fungi). In the cheese rind, PDO explained 63.5% (resp. 47.7%) of the variance for richness and 59.6% (resp. 70.1%) of the dispersion of beta-diversity for prokaryotes (resp. fungi), respectively (Fig. 5A and B; Supplementary Fig. S8; Supplementary Table S6). After PDO, the main driver of the alpha- and beta-diversity of the cheese microbiota was the technological family, followed by variables laid down in the PDO specifications, such as the French region, ripening time, topography, and the dairy species (Fig. 5A and B; Supplementary Fig. S8; Supplementary Table S6). As for cheese production practices, rind care practices, the use of wooden boards for cheese ripening, the salting method, the relative humidity of the ripening room, and the pH in cheese at the end of ripening were among the most influential drivers of the diversity and composition of cheese microbiota. These variables were significant for both the bacterial and fungal communities in the core and rind.

**Figure 6:**
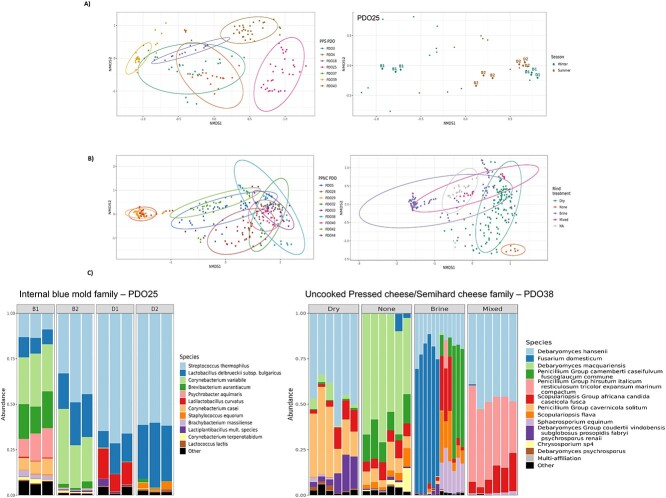
Environmental, farming and technological parameters contributing to cheese microbiota shaping at technological family level in PPS (internal blue mold) and PPNC (uncooked pressed/Semihard) cheeses. (A) Main contributors to bacterial community shaping on the surface of PPS (internal blue mold) cheeses comprising seven PDOs (*N* = 155). NMDS ordinations based on Bray–Curtis dissimilarities. PERMANOVA analyses were performed to test the effects of PDO within the PPS (internal blue mold) family (left panel) (R2 = 0.578, *P*-value < .001) and of the season on the PDO25 blue cheeses (right panel) (R2 = 0.112, *P*-value < .010). (B) Main contributors to fungal community shaping on the surface of PPNC (uncooked pressed/semihard cheese) comprising nine PDOs (*N* = 271). NMDS ordinations based on Bray–Curtis dissimilarities. PERMANOVA analyses were performed to test the effects of PDO within the PPNC (uncooked pressed/semihard cheese) family (left panel) (R2 = 0.590, *P*-value < .001) and of the rind treatment (right panel) (R2 = 0.270, *P*-value < .001). Dry: dry surface rubbing. None: no rind treatment. Brine: Brine treatment by wiping, dipping, or spraying. Mixed: combined treatments according to the ripening stage. (C) Left panel: bacterial profiles in PPS (internal blue mold) PDO25 individual cheeses from producers B (farmhouse cheese produced from raw milk) and D (cheeses produced from commingled, thermized milk) sorted according to the season. B1, D1: winter productions. B2, D2: summer productions. Right panel: fungal profiles in PPNC (uncooked pressed/semihard cheese) PDO38 individual cheeses sorted according to the rind treatment (R2 = 0.650, *P*-value < .001).

Within every cheese technological family studied, the PDO and the French region were significant drivers of the bacterial and fungal communities, except for the bacterial community in the core of the PLCL (lactic washed rind) cheeses (Fig. 5A and B; Supplementary Fig. S8; Supplementary Table S6). When analyzing the alpha- and beta-diversity indices to identify technological practices that might contribute to shaping the cheese microbiota by PDO within each cheese family, several PDO-dependent or cheese production practices, such as the dairy species, were excluded because they were conserved among PDOs within the cheese family considered. Whenever tested, the effects of the season, type of production (farmhouse or corporate dairy), and milk treatment on the shaping of microbial communities varied considerably according to the cheese technology family, with different outcomes for the bacterial and fungal communities: two of the outstanding examples are illustrated in Fig. 6. Within the PPS (internal blue mold) cheese family, analysis of the Bray–Curtis dissimilarity matrices of bacterial communities on the surface of PDO25 cheeses showed that the 24 farmhouse cheeses produced from raw milk differed according to the season, as illustrated by the species-level compositions of cheese productions B1 and B2 (Fig. 6A and C). On the other hand, the season did not significantly affect the 12 cheeses produced from commingled and thermized milk. For the PPNC (uncooked pressed/semihard cheese) family, which includes nine PDOs for a total of 543 cheese samples analyzed, the PDO and rind treatment explained 59% and 27%, respectively, of the dispersion observed for the beta-diversity of fungal communities on the cheese surface (Fig. 6B). More specifically, for PDO38, the rind treatment explained 65% of the observed dispersion, with cheeses that underwent no rind treatment being mainly differentiated by an increased relative abundance of ASVs assigned to *Debaryomyces macquariensis* (Fig. 6C, right panel).

In conclusion, for milk samples on the one hand and cheese samples on the other, the alpha- and beta-diversity descriptors of the microbiota were strongly and primarily influenced by the PDO and PDO-associated variables.

### A high proportion of French PDO cheese taxa originated from milk

**Figure 7:**
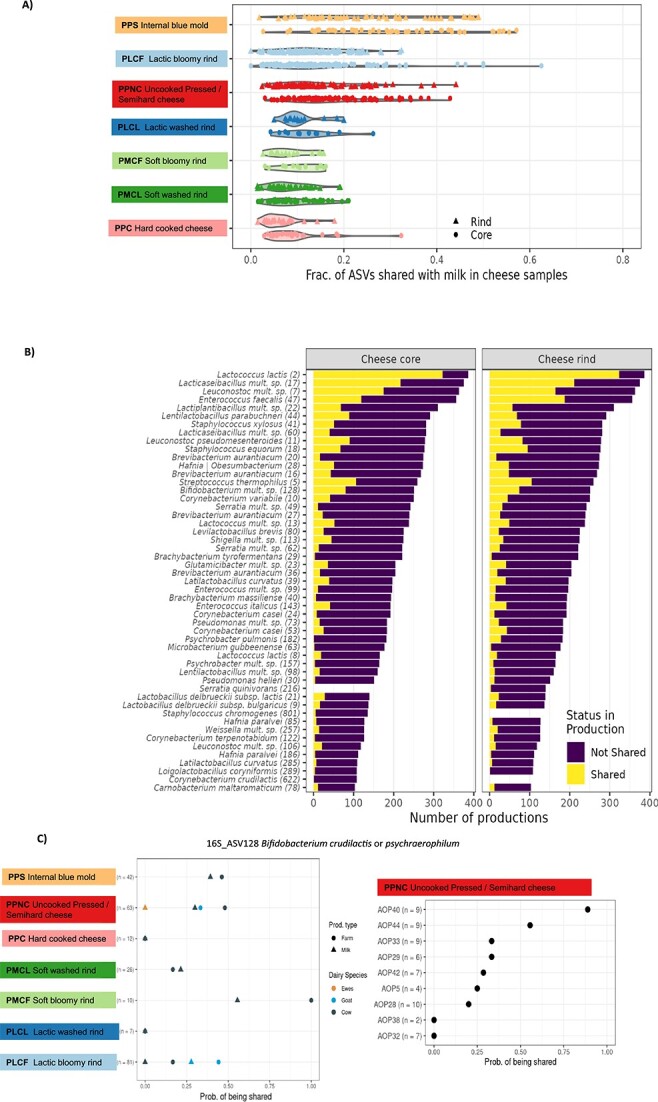
Shared bacterial ASVs between milk and cheese bacterial microbiota at the individual level. (A) Contribution of milk bacterial ASVs to cheese bacterial microbiota according to the cheese family. The ASVs shared between a given milk sample and the derived cheese were identified, i.e. a total of 740 milk-cheese core or milk-cheese surface pairs. The violin plots display the fraction of bacterial ASVs shared with milk in cheese samples, for each production, according to localization (cheese rind and core) and to cheese families. (B) Bacterial species assigned to the most prevalent shared ASVs in cheeses. The histograms show for each ASV the fraction of the 386 cheese productions in which this ASV was shared with the milk or not shared. The figure has been thresholded to the ASVs detected in at least 100 of the 386 cheese productions. The number in parenthesis next to the species name is the number of the most abundant ASV in that species. (C) Probability of 16S_ASV128 *Bifidobacterium* group *crudilactis psychraerophilum* to be shared with the cheese core. Left panel: probability to be shared according to the cheese families, the dairy species and the production type. Right panel: probability to be shared across the nine PDOs from the PPNC (uncooked pressed/semihard cheese) family.

Of the 820 bacterial and 333 fungal species identified in the cheese dataset, a total of 346 bacterial and 212 fungal species, i.e. 42.2% and 63.6%, respectively, of the bacterial and fungal richness of the cheeses, were also identified in the milk dataset. In order to assess the specific contribution of the milk microbiota to the microbiota of the cheese directly derived from this milk, the microbial taxa shared between milk and cheese samples were paired according to the production batch and identified for each of the productions studied, i.e. a total of 740 milk-cheese core or milk-cheese surface pairs. The ASV ranking was chosen for this analysis as being the most accurate provided by amplicon analysis to assess the contribution of the microbiota of milk to that of the associated cheese. One hundred and forty-five bacterial ASVs and 178 fungal ASVs were shared between paired milk and cheeses, belonging to 116 bacterial and 104 fungal species. On average, milk and cheese from the same production shared 6.58 (±4.47) bacterial ASVs and 16.8 (±5.7) fungal ASVs (Fig. 7A). In cheeses, the shared ASVs represented 15.2% (±10.64%) of bacterial diversity and 41.05% (±12.43%) of fungal diversity. Their cumulative relative abundance reached, on average, nearly 44% and 84% of the bacterial and fungal communities on the cheese rind and nearly 64% and 90% of the bacterial and fungal communities in the cheese core, respectively. Bacterial shared ASVs were more abundant in the cheeses’ core than on the rind, with significant variations according to the technological family (Fig. 7A). The qualitative importance of shared ASVs in cheese microbiota varied considerably across cheese families, with PPC (hard cooked cheese) hosting the lowest fraction of bacterial and fungal shared ASVs (Fig. 7A). PPS (internal blue mold) cheeses shared the largest and most variable fraction of their diversity with milk, representing on average 30% (min. 2.6% − max. 57%) and 26% (min. 1.8% − max 46%), respectively, of core and surface bacterial diversity, and 48.7% (min. 26.5% − max. 71.8%) and 51% (min. 19.7% − max 82.3%), respectively, of core and surface fungal diversity.

Figure 7B details the species assigned to the most prevalent bacterial and fungal shared ASVs in cheeses (limited to those detected in at least 100 of 386 productions). *Lactobacillales* and *Micrococcales* accounted for 42.7% and 26.5% of bacterial ASVs shared between milk and cheese core or surface, respectively. A total of 23 bacterial ASVs detected in both milk and associated cheese were attributed to species of the genera *Brachybacterium*, *Brevibacterium*, *Glutamicibacter*, *Lactococcus*, *Lactobacillus*, *Lacticaseibacillus*, *Lactiplantibacillus*, *Leuconostoc*, *Staphylococcus*, and *Streptococcus*. They could originate either from strains introduced as starter cultures or from wild strains with identical ASV sequences. The ASV2 assigned to *Lactococcus lactis* was shared between milk and cheese in 84% of productions. Among the 122 shared bacterial ASVs not known to be introduced as starters or ripening cultures in the productions studied, ASV47 assigned to *Enterococcus faecalis* was shared in 48.4% of cheeses on the surface and 30.8% in the core. ASV128 *Bifidobacterium crudilactis* or *psychraerophilum* and ASV28 *Hafnia alvei* were shared in 20.7% and 13.2% of the cheese cores, respectively. Fifteen fungal ASVs were shared between milk and the associated cheese in more than 50% of the cheese productions, of which 10 ASVs were assigned to species not known to be added as ripening starters in these productions, including four species of the genus *Candida*, such as ASV19 *C. zelanoides*, ASV5 *Diutina catenulata*, ASV6 *Debaryomyces coudertii*, and ASV12 *Yarrowia lipolytica*.

Parameters related to milk production management, such as the dairy species, type of production (farmhouse vs. commingled milk), and cheese technology, were identified as contributing to the large variability in the fraction and composition of ASVs shared between milk and cheese. 16S_ASV128 *Bifidobacterium crudilactis* or *psychraerophilum* was detected exclusively in goat’s and cow’s milk and in cheeses derived from these two dairy species. It was differentially shared between milk and cheeses across cheese families (*P* < .01, linear regression), mainly in PLCF (lactic bloomy rind) (shared in 45/81 = 55.5% of productions), followed by PPS (internal blue mold) (19/53 = 35.8%) and PPNC (uncooked pressed/semihard cheese) (16/63 = 25.4% of productions), with no sharing in PPC (hard cooked cheese) and PLCL (lactic washed rind) (Fig. 7C). It was mainly enriched in PPNC (uncooked pressed/semihard cheese) PDO40 cheeses (average 4.2% relative abundance in cheese core), which constituted a signature. ITS_ASV19 *C. zelanoides* was most prevalent and abundant in cow’s and sheep’s milk and in cheeses derived from these two dairy species. It was shared between milk and cheeses in all sheep-derived productions (18 productions) but was more enriched in PPNC (uncooked pressed/semihard cheese) PDO38 (average 4.9% relative abundance in cheese core) than in PPS (internal blue mold) PDO39 (0.07%) (Fig. 7D).

## Discussion

This study highlighted that PDO cheeses and milk harbored many more microbial species than the microbial cultures deliberately introduced into dairy products (95 bacterial and 40 fungal species; Bulletin of the IDF N° 495/2018, [31]), thus showing that indigenous species are probably responsible for the typicality of PDO cheeses.

The 2400 PDO cheeses and 370 PDO milks were not equivalent in terms of their alpha diversity levels, as the mean number of bacterial (resp. fungal) ASVs per sample ranged from 44 for cheese (resp. 172) up to 2160 for milk (resp. 4786). Various other studies have reported such a variability but with lower alpha diversity values [1, 11, 12, 32–35]. The PDO milk and cheese cores presented similar levels of bacterial and fungal diversity. By contrast, the PDO cheese rinds had much higher bacterial than fungal diversity, which was in line with the results obtained by Raymond-Fleury *et al.* [33] but contrasted with those of Wolfe *et al.* [4]. This may have been due to the latter working at the genus level, where differences between bacteria and fungi are less pronounced, whereas we worked at the ASV level.

Our results revealed the important role played by ruminant species in the milk microbiota, in line with the conclusions of previous studies in the literature [36]. Another result of our study indicated how the cheese family contributed to the cheese microbiota. In all the PDO cheeses studied, a key feature was the absence of core microbial species. At the cheese family level, a core microbiota was identified as being mainly composed of species that were potentially added as starters for cheese making and ripening. Nevertheless, our network structure analysis suggested that different sub-dominant species alternated in their contribution to structuring the cheese microbiota. Overall, these results highlighted that PDO cheese microbiota differed in terms of richness and composition not only between the seven cheese families, but also, for the first time, between the PDOs of a given cheese family.

Many geographical and technological factors may contribute jointly to shaping the milk and cheese microbiota depending on the PDO. It may be challenging to distinguish the specific effects of these factors on milk and cheese microbiota because of the expected interactions between farming practices, geographical factors, and the strong structuring, by PDO constraints, of technological factors.

In the case of cheese microbiota, the effects of geographical factors and processing practices have been the subject of numerous studies in the past, sometimes reaching contradictory conclusions [1, 4, 11, 37]. In our study, we found that the French region, topography, season, processing practices, and the PDO-specific know-how used to mature the cheeses (ripening time, rind treatment, use of wooden supports, and salting methods) all contributed to the richness and composition of the cheese microbiota, with different outcomes depending on the technological family of the cheese. To identify the existence of a biogeography of the cheese microbiota, the results of our study also underline the importance of using a typology based on significant steps in the cheese-making process and of analyzing geographically distant cheeses within each technological family.

A particularly original result of our study is to show that the specific characteristics of PDO microbiota were shaped from the milk onwards. Indeed, PDO was the second most important contributor to the structuring of the milk microbiota after the dairy species. This finding was in line with the results of the study by Bokulich *et al.* [8], who showed that geography and dairy ruminant species influenced the microbiota of Matsoni fermented milk. In addition, we showed that several factors specified by the PDO, including geographical factors, such as the French region and topography and farming practices such as animal breed and feed, significantly influenced the richness and composition of the milk microbiota. The latter factors are therefore thought to jointly contribute to the specific microbial composition of milk from each PDO. The specific effect of the season on the richness and composition of cow’s milk microbiota was minor when compared with that of udder hygiene and animal housing conditions (indoor vs. outdoor, type of barn, bedding conditions), the seasonality of which varied from farm to farm. Overall, these findings were in accordance with a large body of studies that have highlighted the influence of farming practices, such as milking hygiene [14, 38–42], season [32, 43–46], lactation stage [46, 47], or the combined effect of multiple farming practices on the bovine milk microbiome [48–51]. Studies investigating the determinants of fungal communities in the milk of dairy ruminants, as well as the milk microbiome of small ruminants (goat and sheep), are much rarer [52, 53]. To our knowledge, the structuring role of PDO on cattle milk microbiota had never previously been demonstrated. The effects of region and topography observed in our study were in line with the results of Italian studies, which showed that the bacterial profile of raw cow’s milk from Italian alpine pasture differed from that of raw milk from lowland areas, in particular regarding an increased relative abundance of *Bifidobacterium crudilactis* [54–56]. By contrast, in 355 raw cow’s milk samples collected from five farms across vast Chinese geographic regions, the bacterial profiles were highly diverse according to seasonality but not sampling region [44]. Large-scale studies of milk produced in different regions and with finely characterized practices are needed to elucidate the microbial biogeography of milk.

To our knowledge, we provided the most exhaustive study to date on the relationship between milk and cheese microbiota at the batch level. We thus revealed the ASVs shared between each milk and its associated cheese for a total of 740 milk-cheese pairs. ASV ranking was chosen for this analysis because it is the most accurate provided by amplicon analysis to assess the persistence of milk-derived microbial DNA in cheese. However, this approach is limited by the low microbial load of the milk, and prior freezing of whole milk may have resulted in lysis of some cells, which would be lost during subsequent centrifugation for DNA extraction. These limitations may have hampered the detection of the DNA of sub-dominant populations in the milk [57, 58], although they may be enriched in the cheese. In addition, due to its lower level of resolution compared with whole-genome approaches, this approach does not allow us to confidently conclude that an ASV detected in a cheese is derived from the same microbial strains detected in the milk. Likewise, it does not differentiate between wild populations and those introduced as starter cultures. Nor does it provide any information on the viability of the microbial cells at the origin of these ASVs. Despite these limitations, this study has provided new insight into the potential of milk as a source of fungal diversity, and particularly yeasts, for cheese. These findings are consistent with those obtained using Robiola di Roccaverano PDO (PMCF Soft bloomy rind) cheese from the Piedmont region of Italy [59]. Each French PDO cheese shared a large proportion of its fungal diversity with the milk from which it was derived (on average 41.05%) compared with an average of 15.2% of its bacterial diversity. The most prevalent shared bacterial ASVs across cheeses included lactic acid bacteria and ripening bacteria among the species known to be deliberately added as starter cultures. However, 84% of the shared bacterial ASVs were assigned to genera or species not listed as starter cultures. Overall, the analysis of shared ASVs confirmed that milk was a reservoir for microorganisms of recognized technological interest in cheese, such as lactic and ripening bacteria and certain fungal species. This analysis also emphasized milk as a source of microbes that could constitute the defining microbial features of technological families or PDOs. However, their potential technological properties remain largely unexplored, such as ASV128 (*Bifidobacterium*) and ASV19 (*C. zelanoides*) in PDO40 and PDO38 cheeses, respectively. The *Bifidobacterium* genus was detected in 30.3% of the cheese samples, close to the 33% found across 21 samples from the most common Italian raw milk cheeses [60]. Our results also revealed that 20.7% of the cheeses shared an identical ASV128 assigned to *Bifidobacterium crudilactis* with the milk from which they were derived. This was in line with the conclusions reached by Milani *et al.* [61] that milk-derived bifidobacterial taxa consistently contribute to the bacterial biodiversity hosted by Parmesan cheese. With a relative abundance of the *Bifidobacterium* genus ranging from 0.02% to 11.05%, PDO40 cheeses stand out from French and Italian PDO cheeses. These microbiological traits could be linked to the peculiarities of PDO40 cheese insofar as it is produced exclusively from the milk of cows grazing on mountain pastures [55, 56], which is then collected and curdled in wooden vats.

The influence of geographical factors on the structuring of PDO milk and cheese microbiota could be explained by coalescence with microbiomes in the environment of dairy ruminants (pastures, water, air, cow feces, etc.) [14, 15, 52, 61, 62], as well as in cheese dairies (brine, wooden vats) [63, 64] and maturing cellars [65], or even in the landscape and urban environment of the dairy facilities [66]. All these factors make up a unique combination at the local level, shaping the environmental microbial exposome for milk and cheese, which can have an impact on the taxonomic composition and final organoleptic characteristics of cheese at each production site [1, 67–69].

In conclusion, we found that PDO cheeses belonging to the same family but originating from different regions do share several core microbial taxa. In this respect, our results agree with the findings of Wolfe *et al.* [4]. However, in light of the fine-grained cheese typology used in our study, we also found that the microbiota in the milk and cheese of each PDO had a specific composition. These compositions were significantly influenced by a combination of geographical factors and human practices that underpin the specific characteristics of each PDO and foster the link between PDOs and their “terroir.” Moreover, network analysis revealed possible community interaction patterns that can be tested in targeted experiments. Strategies combining amplicon sequencing with chemical and physical reference methods have shown potential for the authentication of PDO cheeses [70]. With this in mind, the 2316 microbial profiles of cheese core and rind obtained during this study will serve to initiate an exhaustive and unique repository of French PDO cheeses and the associated practices, to be expanded in the future with a broader panel of cheeses produced worldwide. Our results highlighted the importance of considering the milk-cheese continuum in a microbial biogeographical analysis of cheeses. This will require the implementation of multi-omics and integrative approaches that also consider the biochemistry of milk and cheese [3, 71]. Our results will support PDO cheese sector stakeholders in their commitment to maintaining indigenous microbial diversity along the milk-cheese continuum, when defining the farming and processing specifications for each PDO. Traditional products, such as PDO cheeses, which are influenced by geographical factors, are particularly vulnerable to the effects of climate change on livestock farming. This cutting-edge knowledge will support policy-makers in their decisions to safeguard highly biodiverse dairy products worldwide.

## Supplementary Material

Suppl_Info_Text_FigS1-S10_TabS2_clean_version_ycae095

Supplementary_TableS1_R1_ycae095

Supplementary_TableS3_R1_ycae095

Supplementary_TableS4_R1_ycae095

Supplementary_TableS5_R1_ycae095

Supplementary_TableS6_R1_ycae095

## Data Availability

Raw reads have been deposited to the European Nucleotide Archive under the project accession number PRJEB64600 for bacterial 16S rDNA amplicons and PRJEB64628 for fungal ITS amplicons. The datasets produced in this study are available in the following database: Recherche Data Gouv under the accession number: https://doi.org/10.57745/UCJG6S. The authors confirm that all supporting data and protocols have been provided within the article or through supplementary data files.
