# Supplementary material for: A comprehensive, large-scale analysis of “terroir” cheese and milk microbiota reveals profiles strongly shaped by both geographical and human factors
Source: ISME Commun. 2024 Jul 11;4(1):ycae095. doi: 10.1093/ismeco/ycae095 (PMC11301721; doi:10.1093/ismeco/ycae095)
Supplement: Suppl_Info_Text_FigS1-S10_TabS2_clean_version_ycae095 [file suppl_info_text_figs1-s10_tabs2_clean_version_ycae095.docx]

**Supplementary Informations**

**A comprehensive, large-scale analysis of ‘terroir’ cheese and milk microbiota reveals profiles strongly shaped by both geographical and human factors**

Françoise Irlinger^1^, Mahendra Mariadassou^2^, Eric Dugat-Bony^1^, Olivier Rué^2^, Cécile Neuvéglise^3^, Pierre Renault^4^, Etienne Rifa^5^, Sébastien Theil^5^, Valentin Loux^2^, Corinne Cruaud^6^, Frederick Gavory^6^, Valérie Barbe^6^, Ronan Lasbleiz^7^, Frédéric Gaucheron^8^, Céline Spelle^7^, Céline Delbès^5*^

^1^ Université Paris Saclay, INRAE, AgroParisTech, UMR SayFood, 91120 Palaiseau, France

^2^ Université Paris-Saclay, INRAE, MaIAGE, 78350, Jouy-en-Josas, France

^3^ UMR SPO, Université Montpellier, INRAE, Institut Agro, Montpellier, France

^4^ Université Paris-Saclay, INRAE, AgroParisTech, Micalis Institute, 78350 Jouy-en-Josas, France

^5^ Université Clermont Auvergne, INRAE, VetAgro Sup, UMR 545 Fromage, Aurillac, France

^6^ Genoscope, Institut François Jacob, Commissariat à l’Energie Atomique (CEA), Université Paris-Saclay, 91057, Evry, France

^7^ CNAOL, Maison du Lait, 75009 Paris, France

^8^ CNIEL, Maison du Lait, 75009 Paris, France

*Corresponding author: Céline Delbès, [celine.delbes@inrae.fr](mailto:celine.delbes@inrae.fr)

**Supplementary Methods**

**DNA extraction**

A 250-mg aliquot of cheese sub-samples was added to a 2-ml tube containing 350 mg of zirconium beads (diameter, 0.1 mm; Sigma, St-Quentin-Fallavier, France), 250 µl of guanidine thiocyanate (4 M) in Tris-HCl (0.1 M, pH 7.8) and 40 μl N-lauryl sarcosine (10%) and shaken in a Precellys Evolution bead beater (Bertin, Montigny-le-Bretonneux, France) for 20 sec at a speed of 6 500 m/s. 75 μl of lysozyme (3mg) and lyticase (100 unity) were added, and the tube was incubated for 30 min in a water bath at 37°C. 40 μl of proteinase K (15 mg/ml) and 100 μl of sodium dodecyl sulfate (20%) were then added. The tube was incubated for 30 min in a water bath at 55°C. 200 μl of sodium phosphate buffer (0.1 M, pH 8), 200 μl of 50 mM acetate-10 mM EDTA buffer (pH 5), and 500 μl of phenol-chloroform (25:24:1, pH 8) were then added. The tube was vigorously shaken in a Precellys Evolution bead beater (Bertin, Montigny-le-Bretonneux, France) for 45 sec at 10000 m/s. The tube was incubated for 2 min at 55°C and cooled on ice for 5 min. After another 45 seconds of mixing 10000 m/s), the tube was incubated for 2 min at 70°C, cooled on ice for 5 min, and centrifuged for 20 min at 14,000 rpm and room temperature, separating two phases. The upper aqueous phase was recovered in a 2-ml tube (Phase Lock Gel Heavy; Eppendorf, Hamburg, Germany) and mixed with 500 µl of phenol-chloroform (25:24:1, pH 8). The tube was centrifuged for 5 min at 14,000 × rpm and 20°C. Chloroform (500 µl) was then added to the tube, which was mixed gently. After a third centrifugation, the aqueous phase was recovered, mixed with two μl of RNase A (20 mg/ml; SERVA Electrophoresis GmbH, Heidelberg, Germany), and incubated for 30 min at 37°C. The extracted DNA was then purified and concentrated with the Genomic DNA Clean & Concentrator-10 kit (Zymo Research). A blank extraction (adding nothing to the bead tube) was performed alongside sample extractions for the negative extraction controls. DNA extracts were stored at −20 °C until further analysis.

To extract DNA from milk, we thawed 180 mL samples in a water bath at 25°C, then 12 mL of SDS (sodium dodecyl sulfate 20% solution) were added to 120 mL of milk, which were then incubated for 30 min at 30 °C. After centrifugation (5300× g, 30 min, 4 ◦C), the fat layer and the supernatant were removed. The pellets obtained were mixed with 1 mL sterile PBS (Phosphate-buffered saline), incubated for 10 min at 30°C, centrifuged (13,000× g, 5 min, 4 ◦C) and stored at −20 ◦C. Total DNA extraction was then performed as described for cheese samples.

**PCR amplification**

Two primer pairs were used to amplify two ribosomal RNA (rRNA) barcode loci in a 20 cycle PCR: a 16S rRNA gene fragment targeting the V3-V4 regions to characterize bacterial diversity was amplified using the primers 16S_V3F (5′-ACGGRAGGC WGCAGT-3′) and 16S_V4R (5′-TACCAGGGTATCTAATCCT-3′), while a fungal gene fragment targeting the ITS2 region to characterize fungal diversity was amplified using the primers ITS3f (5′- GCATCGATGAAGAACGCAGC -3′) and ITS4_KYO1 (5′- TCCTCCGCTTWTTGWTWTGC -3′). The PCR products were then purified using a MinElute PCR purification kit (Qiagen, Courtaboeuf, France) and quantified using the QuantiFluor staining kit (Promega, USA). A second PCR of 7 cycles was then duplicated for each sample under similar PCR conditions, with purified PCR products as a matrix (7.5ng of DNA were used for a 25μl mix of PCR) and dedicated fusion primers (‘F479/AdaptorB’, ‘R888/MID/AdaptorA’) integrating needed adaptors, keys and multiplex identifiers at 5’ extremities. All duplicated PCR products were then pooled, purified using a MinElute PCR purification kit (Qiagen, Courtaboeuf, France), and quantified using the QuantiFluor staining kit (Promega, USA).

For all libraries, equal amounts from 352 samples (4 plates) were indexed, pooled, and then cleaned to remove excess nucleotides, salts, and enzymes using the Agencourt AMPure XP system (Beckman Coulter Genomics). 100μl of TE buffer (Roche) was used for the elution. Sequencing was then carried out on the Illumina HiSeq 2500 platform (2x250) by Genoscope (Evry, France).

**Quality control of the sequences and bioinformatics analyses**

The resulting sequences were analyzed using workflow combining dada2 v.1.16 and FROGS 3.2.2 software. For ITS and 16S data, adapters were removed from reads with cutadapt (v1,12), and reads were filtered with the *filterAndTrim* function from the dada2 package (truncQ=2, minLen=50, rm.phix=TRUE, maxN=0 and maxEE=c(2,2). Then, the remaining sequences were dereplicated with the *derepFastq* function, and errors were corrected with both *learnErrors* and *dada* functions. 16S paired-end reads were merged with *the mergePairs* function, and only merged sequences were kept. For ITS paired-end reads, unmerged reads were conserved. Then, for both datasets, chimera were removed with *the removeBimeraDenovo* function. Finally, amplicon sequence variants (ASVs) were affiliated with the FROGS *affiliation_OTU* tool with silva v.132 and DAIRYDB v1.1.2 databanks for 16S data, and with UNITE v7.2 and a collection of personal sequences. For the most abundant and prevalent ASVs, the taxonomic affiliation was manually checked and corrected when needed. More precisely, ASVs were blasted against different databases (e.g., NCBI, YEASTIP...) to confirm or correct the affiliation, and we removed some ASVs (remaining chimera, contaminations). When taxonomic resolution at the species level was impossible (identical sequences between two or several species), we defined species groups and labeled ASVs accordingly.

Prevalence and abundance-based filters were applied to the ASV set to take advantage of the experimental design of our dataset. First, all ASVs with a specificity value in the negative controls higher than 0.7 were removed. For cheese samples, bacterial ASVs were first analyzed at the PDO level and flagged as genuine if they satisfied any of the following criteria : (i) present in all rind (resp. core) samples of cheeses from a batch, (ii) present in at least 40% of all rind (resp. core) samples from the PDO, (iii) present in at least 50% of the samples (rind of core) from the PDO, (iv) present in at least 70% of all batches (in either a core or a rind sample), (v) relative abundance higher than 5e-5 in at least one batch (in either rind or core samples). The final cheese ASV set was consolidated by keeping ASVs genuine in all PDOs or the 12 most abundant ASVs of any PDO. For fungal ASVs, we applied the same rule but filtered out all ASVs with a confidence score for Fungi lower than 50% (as computed by IDTAXA on the UTOPIA database). For milk samples, since the starting material had a lower bacterial load and we had only one sample per batch, we kept all ASVs that passed the filters in the cheese samples and all ASVs with more than ten reads and which were either (i) present in at least five samples or (ii) had relative abundance higher than 10% in at least one sample.

***Statistical methods***

Statistical analyses were performed using R (v 4.3.1) with specialized packages: phyloseq (v. 1.34), PLN models (v1.0.1), and custom scripts [26, 27]. Data were rarefied to the same depth before computing alpha and beta diversity indices but not for the network analyses.

The species with >90% prevalence when the detection threshold is set to 0.1% relative abundance were identified as core species. In order to analyse the potential correlation between cheese pH and salt content values and the presence or absence of core microbial species in the cheeses, the relative abundances of the core species have been computed by averaging them across all replicates of a unique production x location (cheese core or rind) x PDO combination. The Spearman correlation was then computed to assess the significance of potential associations.

Alpha-diversity analyses were performed on the observed ASV richness. We compared the diversity across technological families (for cheese samples) or dairy species (for milk samples) using a one-way analysis of variance (ANOVA). Beta diversity analyses were performed on the Bray-Curtis distances, computed separately for the bacterial and fungal fractions. A Nonmetric Multidimensional Scaling (NMDS) was performed on each distance matrix to represent the samples on the principal plane and identify influencing factors. Several factors' impacts on the microbiota were assessed using permutational multivariate analysis of variance (PERMANOVA), as implemented in the adonis2 function from the vegan package.

For microbial transfer indicator determination, the production batches were filtered to keep only those for which milk, core, and rind samples were available. All core and rind replicates from a batch were aggregated by summing their counts. The milk, rind, and core samples were rarefied (independently for each batch) to the same depth to ensure equal detection probabilities in all compartments. For each batch, we computed the number of ASVs shared between the milk and the core (resp. the rind), their fraction among the core (resp. rind) ASVs, and their cumulated relative abundance among the core (resp. rind) microbiota. For all ASVs present in at least 100 cheese rind batches or 100 cheese core production batches, we also computed the number of batches where the ASV was detected and the number of batches shared between the milk and the core (resp. rind). Finally, for a few selected taxa and each unique combination of technological family, production type, and dairy species, we computed the probability of being shared as the fraction of batches in which the ASV was shared among the batches where it was detected in that combination. The effect of the three factors on the probability of being shared was estimated using a logistic regression with simple effects and no interactions.

For network-based analysis, rind and core samples (but not milk) were selected to reconstruct a synthetic variable capturing the main structuring factors as a proxy for the ecological niche. For each PDO cheese, production batches were kept if sequencing data were available for the three replicates of rind and core samples for the 16S and ITS2 abundance tables (386 batches total). The three replicates were merged, and the two resulting relative abundance tables (16S and ITS2) were rarefied to normalize the sequencing depth across batches. A Bray-Curtis distance matrix was then computed for each marker (16S and ITS2), and the two matrices were averaged to create a global distance matrix. Hierarchical clustering of the samples was performed using the ward linkage and identified 5 clusters (Supplementary FigureS1). Each cluster encompasses similar communities and corresponds to a different synthetic ecological niche. ASVs were then aggregated at the species level, and species showing a high prevalence (either >20% in one cluster or >10% in the whole dataset) were selected for network reconstruction (75 bacterial and 57 fungal species).

Network reconstruction was performed on raw (and not rarefied) abundance tables. Since bacterial and fungal amplicons were sequenced independently, we corrected for differences in sequencing depths independently for the two datasets using the GMPR normalization method (Geometric mean pairwise ratio) [28]. A network with nodes corresponding to bacterial and fungal species was computed using the PLN network function from the R package PLN models (v1.0.1) [27]. PLN network is based on the graphical lasso method, a penalized likelihood regression method that avoids spurious correlations induced by shared ecological niches by controlling for structuring environmental variables such as pH, salinity, etc. The previously constructed clusters were used together with location (core/rind) as proxies for ecological niches. The penalty was chosen using stability selection. In short, networks were reconstructed on several data subsamples, and the penalty was chosen to ensure that the final edges were detected in at least 90% of those networks. Finally, microbial modules were identified using stochastic-block models as implemented in the R package sbm (v.0.4.3). The number of blocks was automatically selected with the ICL criterion.

**Supplementary results**

***Characteristics of French PDO cheeses and milk, and associated metadata***

Seven cheese families were identified according to the classification developed by Almena-Aliste & Mietton [29]: (internal blue mold (PPS); soft bloomy rind (PMCF); lactic bloomy rind (PLCF); lactic washed rind (PLCL; soft washed rind (PMCL); uncooked pressed cheese/semi-hard cheese (PPNC) and hard-cooked cheese (PPC), each including between two and 14 PDO. Within each PDO, cheeses were produced exclusively from the milk of a single animal species (cow, ewe, or goat). Around a hundred covariates providing information on the factors required by the PDO (region, topography, animal species, maturation period, etc.), farming practices (feeding, hygiene, etc.), contingent factors (season, type of production, salting method, etc.) and the characteristics of the milk and cheese (pH, microbial counts, etc.), were determined by the cheese stakeholder at the same time as they collected their cheese and milk (Supplementary Table S1). It is worth noting that PDOs from the same cheese family may be located in regions several hundred kilometers apart, in areas with a unique local topography, soil characteristics, and environmental factors (moisture, temperature, etc.).

***Pre-processing of the sequence dataset***

After pre-processing, 8,066 (3,220 for milk, 4,956 for cheese) amplicon sequence variants (ASVs) corresponding to a total of 596,066,102 high-quality bacterial 16S rRNA V3-V4 gene sequences and 5,590 ASVs (4197 for milk, 1662 for cheese), with a total 707,878,724 high-quality fungal internal transcribed spacer (ITS2) gene sequences, were retained and used for downstream analysis (Supplementary Table S2).

***Sample type (milk, cheese core, or cheese rind) shapes the milk and cheese microbiome***

Dissimilarities between the composition of the microbial community in the milk, cheese core, and cheese rind samples were assessed using a Bray-Curtis distance matrix generated from the ASV abundance table. The nonmetric multidimensional scaling plot clearly showed the clustering of samples according to localization (milk, cheese core, or cheese rind) for both the bacterial and fungal communities (Supplementary Figure S10). For this reason, analysis of the environmental and technological factors influencing the microbiota was conducted separately for the milk and cheese samples.

***Structuration within the metadata set***

Correspondence analysis was applied to the metadata set (Supplementary Table S1) to identify the primary relationships between the environmental and technological parameters that characterized the 386 cheese production batches included in this study.

**Supplementary Tables and Figures**

**Table S2. Sequencing data of the fungal (ITS2) and bacterial (V3-V4) communities from milks and cheeses**

|  | **Milks** | | | | | **Cheeses** | | | | | | | |  | | | | |  | | | | |  | | | | |  | | |  |  |
| --- | --- | --- | --- | --- | --- | --- | --- | --- | --- | --- | --- | --- | --- | --- | --- | --- | --- | --- | --- | --- | --- | --- | --- | --- | --- | --- | --- | --- | --- | --- | --- | --- | --- |
| **Targeted regions** | **V3-V4** | | **ITS2** | | **V3-V4** | | | | **ITS2** | | | |  | | | | |  | | | | |  | | | | |  | | |  |  |  |
| **Number of PDO labels** | 43* | | 43* | | 44 | | | | 44 | | | |  | | | | |  | | | | |  | | | | |  | | |  |  |  |
| **Number of samples** | 390 | | 390 | | 2,308 | | | | 2306 | | | |  | | | | |  | | | | |  | | | | |  | | |  |  |  |
| **Number of samples after trimming and cleaning steps** | 370 | | 370 | | 2291 | | | | 2292 | | | |  | | | | |  | | | | |  | | | | |  | | |  |  |  |
| **Number of trimmed and assembled sequences** | 73,075,816 | | 108,280,731 | | 522,990,286 | | | | 599,597,993 | | | |  | | | | |  | | | | |  | | | | |  | | |  |  |  |
| **Number of ASV sequences (>0.005%)** | 3219 | | 4197 | | 4956 | | | | 1662 | | | |  | | | | |  | | | | |  | | | | |  | | |  |  |  |
| **Number of genus assigned (>0.005%)** | 549 | | 671 | | 289 | | | | 175 | | | |  | | | | |  | | | | |  | | | | |  | | |  |  |  |
| **Number of species assigned (>0.005%)** | 1230 | | 1367 | | 820 | | | | 333 | | | |  | | | | |  | | | | |  | | | | |  | | |  |  |  |
|  | ** no samples were collected for PDO20* | | | | | | | | | |  | | | | | |  | | |  | | | | |  | | | | |  | | | |
|  |  |  | | | | | |  | | |  | | | | | |  | | |  | | | | |  | | | | |  | | | |
| **Nb. ASV sequences (>0.005%)** | **Milks** | | | | | | **Cheeses** | | | | | | | |  | | | | | |  | | | | |  |  |  |  |  |  |  |  |
| **Technological families **** | **Samples number** | **V3-V4** | | **ITS2** | | | **Samples number** | | | **V3-V4** | | **ITS2** | | | |  | | | | | |  | | | | |  |  |  |  |  |  |  |
| **Lactic bloomy rind = PLCF** | 128 | 2320 | | 3604 | | | 781 | | | 485 | | 1521 | | | |  | | | | | |  | | | | |  |  |  |  |  |  |  |
| **Soft bloomy rind = PMCF** | 14 | 731 | | 1042 | | | 132 | | | 1030 | | 241 | | | |  | | | | | |  | | | | |  |  |  |  |  |  |  |
| **Soft washed rind = PMCL** | 46 | 1440 | | 2180 | | | 281 | | | 1473 | | 266 | | | |  | | | | | |  | | | | |  |  |  |  |  |  |  |
| **Lactic washed rind = PLCL** | 14 | 1327 | | 1473 | | | 84 | | | 194 | | 139 | | | |  | | | | | |  | | | | |  |  |  |  |  |  |  |
| **Uncooked Pressed cheese/Semihard cheese = PPNC** | 89 | 2784 | | 3652 | | | 540 | | | 1293 | | 867 | | | |  | | | | | |  | | | | |  |  |  |  |  |  |  |
| **Internal blue mold = PPS** | 52 | 2756 | | 3149 | | | 311 | | | 774 | | 313 | | | |  | | | | | |  | | | | |  |  |  |  |  |  |  |
| **Hard cooked cheese = PPC** | 27 | 1247 | | 2037 | | | 163 | | | 680 | | 324 | | | |  | | | | | |  | | | | |  |  |  |  |  |  |  |
|  | ******** *Technological type : (Figure 1; Almena-Aliste & Mietton, 2014)* | | | | | | | | | | | | | | | |  | | |  | | | | |  | | | | |  | | | |
|  |  |  | | | | | |  | | |  | | | | | |  | | |  | | | | |  | | | | |  | | | |
|  | | | | | | | | | | | | | | | | | | | | | | | | | | | | | | | | |  |

The PDO acronym (Protected Designation of Origin) is a designation that has been granted to certain European cheeses, produced using very specific methods, techniques, and ingredients and specific regions.

**Figure S1:** Hierarchical clustering (ward linkage) of the samples selected for the network-based analysis into five habitats. The distance for the clustering used is the average of the Bray-Curtis distances computed on the bacterial fraction (16S marker) and the fungal fraction (ITS marker).


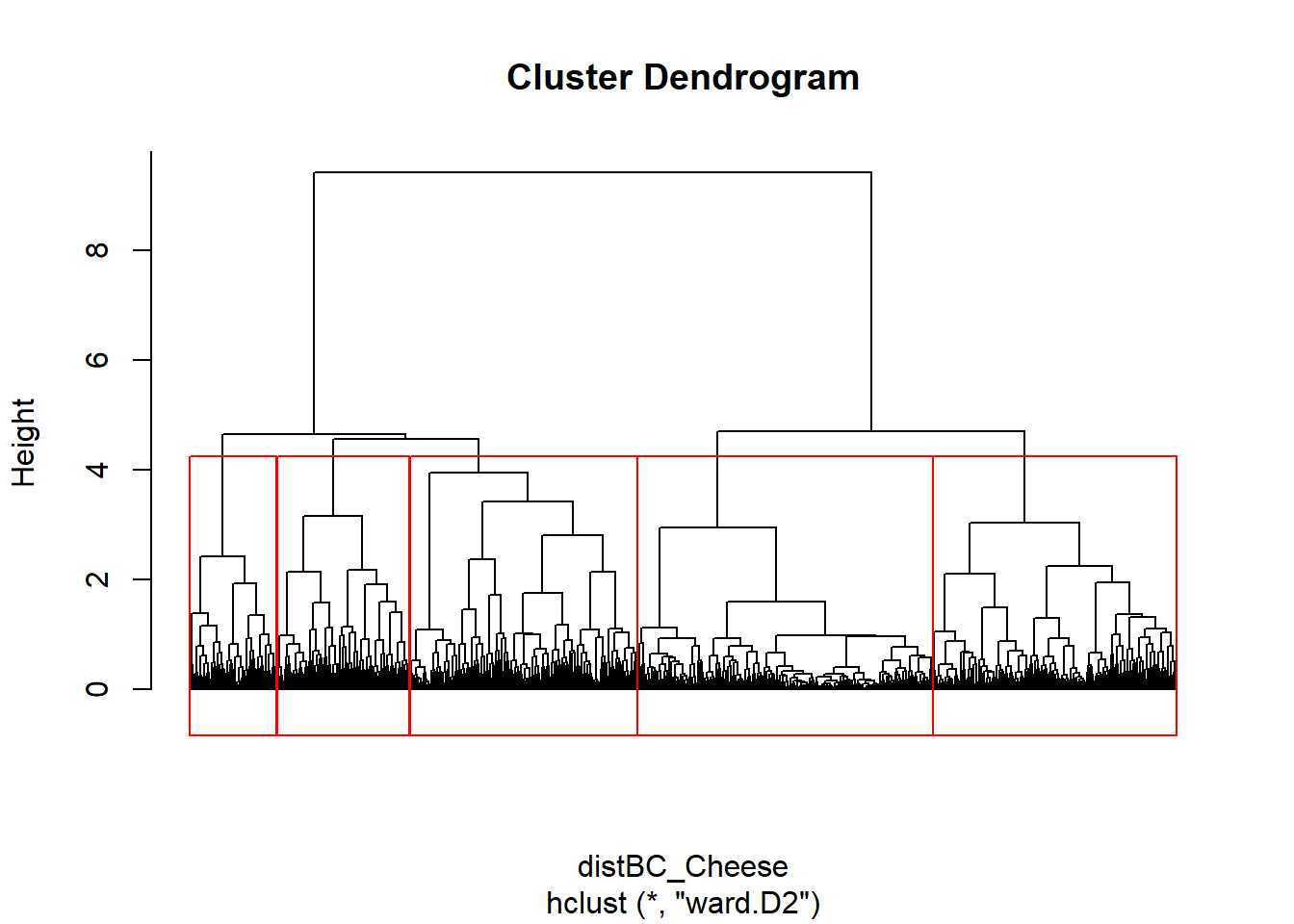


**Figure S2:** Distribution of cheese and milk samples (n= 2702) according to dairy species (A), milk thermal treatment (B), and production type (C). NI stands for Non-Informed.


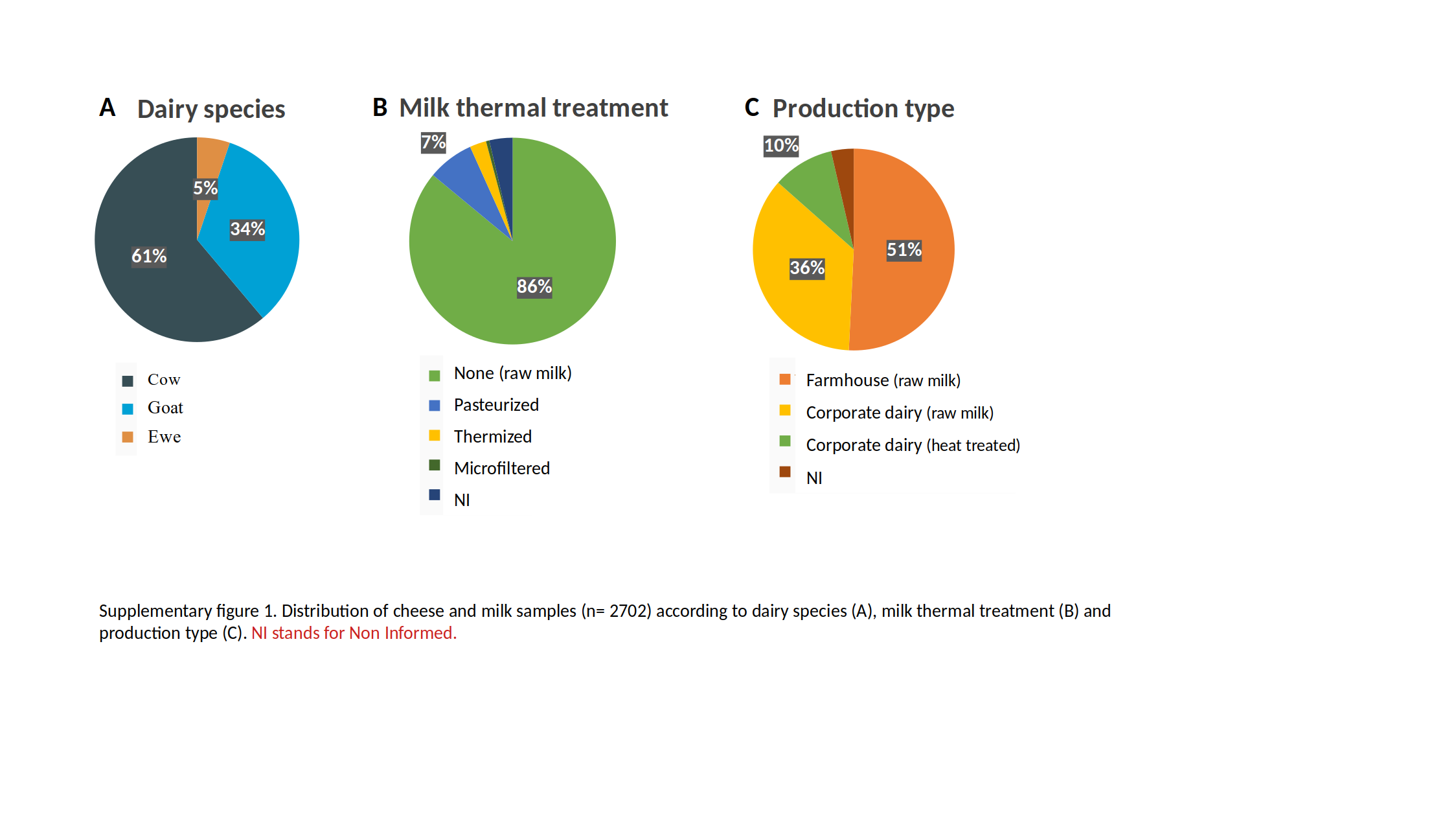


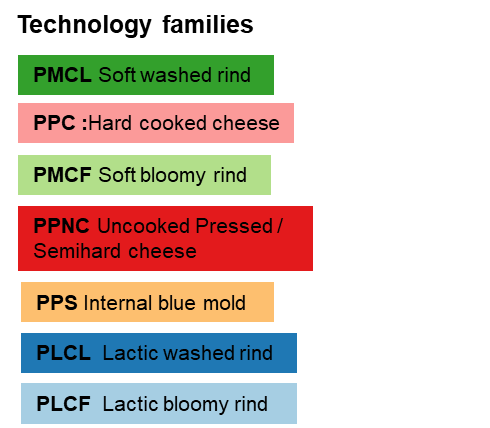

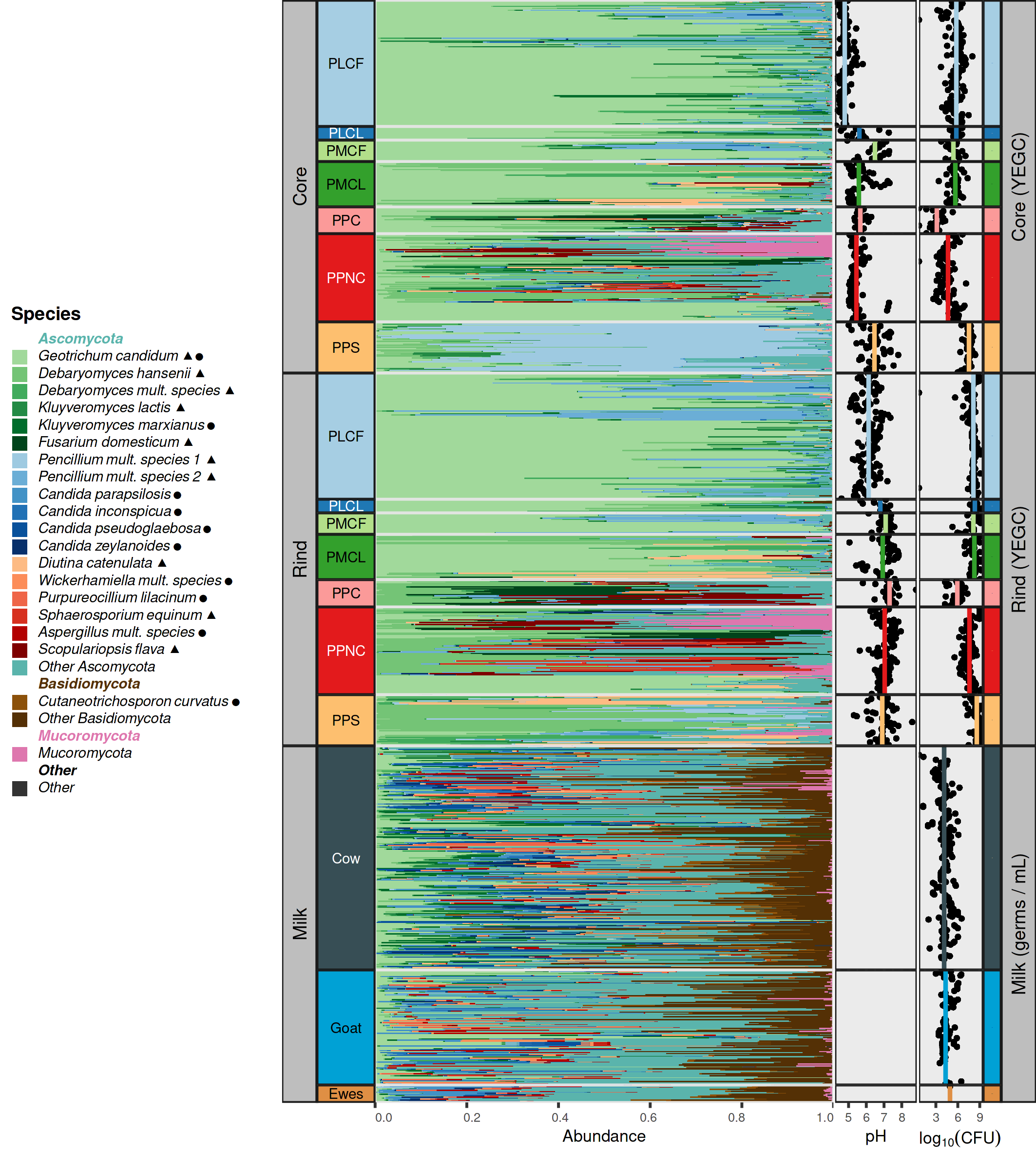


**Figure S3A:** Relative abundance of fungal taxa from milk, and cheeses (core and rind). Milk samples (bottom) are grouped by dairy species, and cheese samples (top) are grouped by technological family. Only the 22 most dominant fungal species are shown in colour; the other subdominant species are grouped into the category “other” (black). Line charts next to each histogram panel show fungal concentrations (on a logarithmic scale) and pH values for each sample. A black dot (resp. triangle) in the legend indicates a species dominant in milk (resp. cheese) samples.


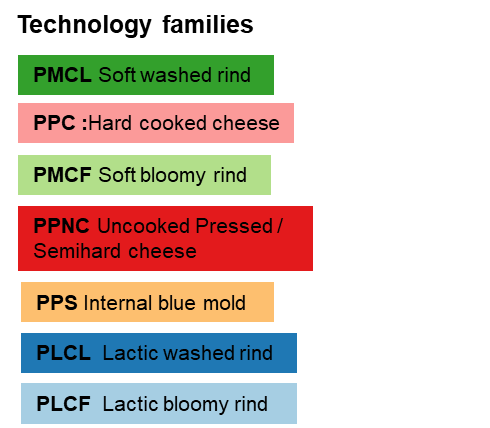

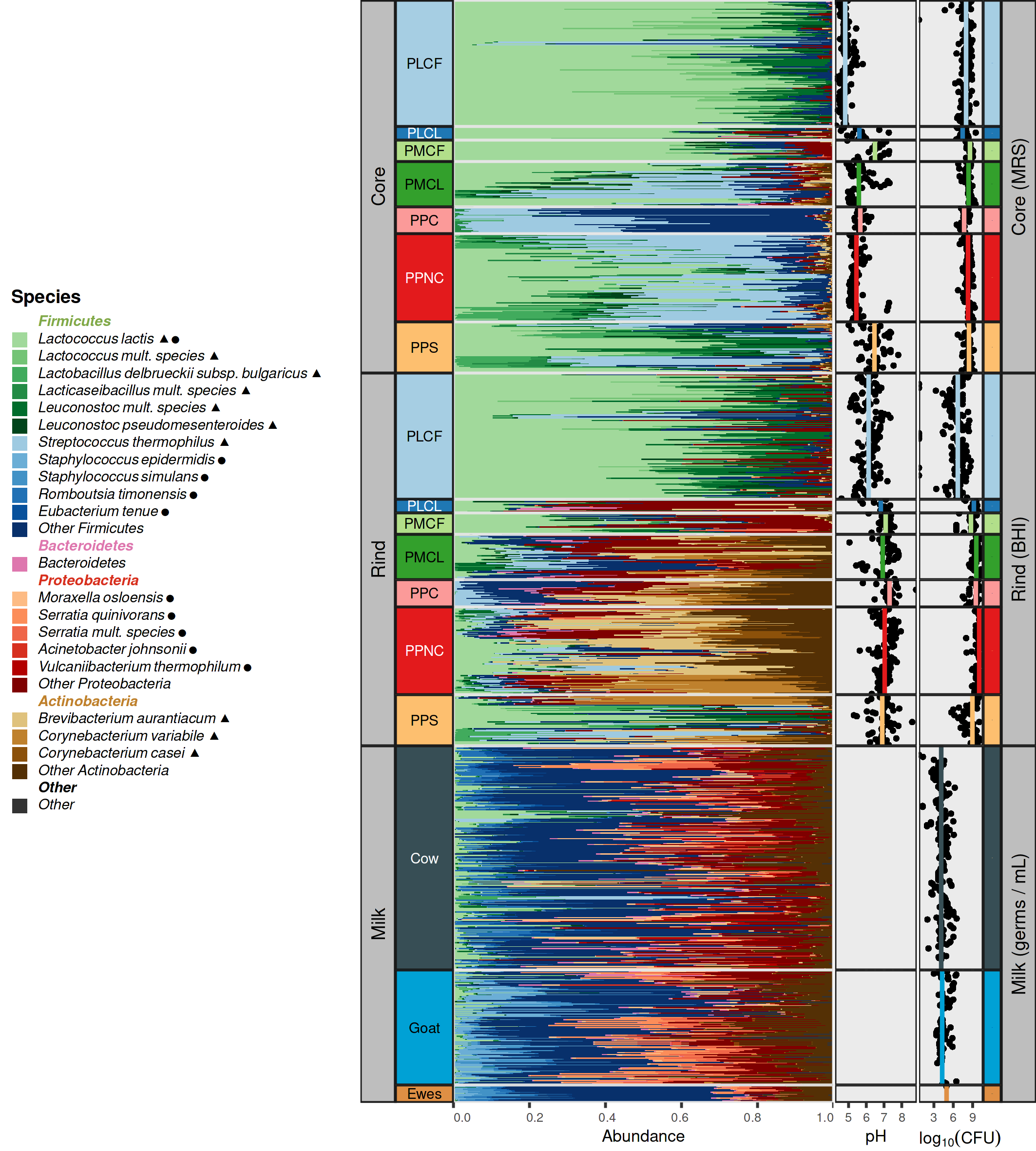


**Figure S3B:** Relative abundance of bacterial taxa from milk and cheeses (core and rind). Milk samples (bottom) are grouped by dairy species, and cheese samples (top) are grouped by technological family. Only the 22 most bacterial fungal species are shown in colour; the other subdominant species are grouped into the category “other” (black). Line charts next to each histogram panel show bacterial concentrations (on a logarithmic scale) and pH values for each sample. A black dot (resp. triangle) in the legend indicates a species dominant in milk (resp. cheese) samples.


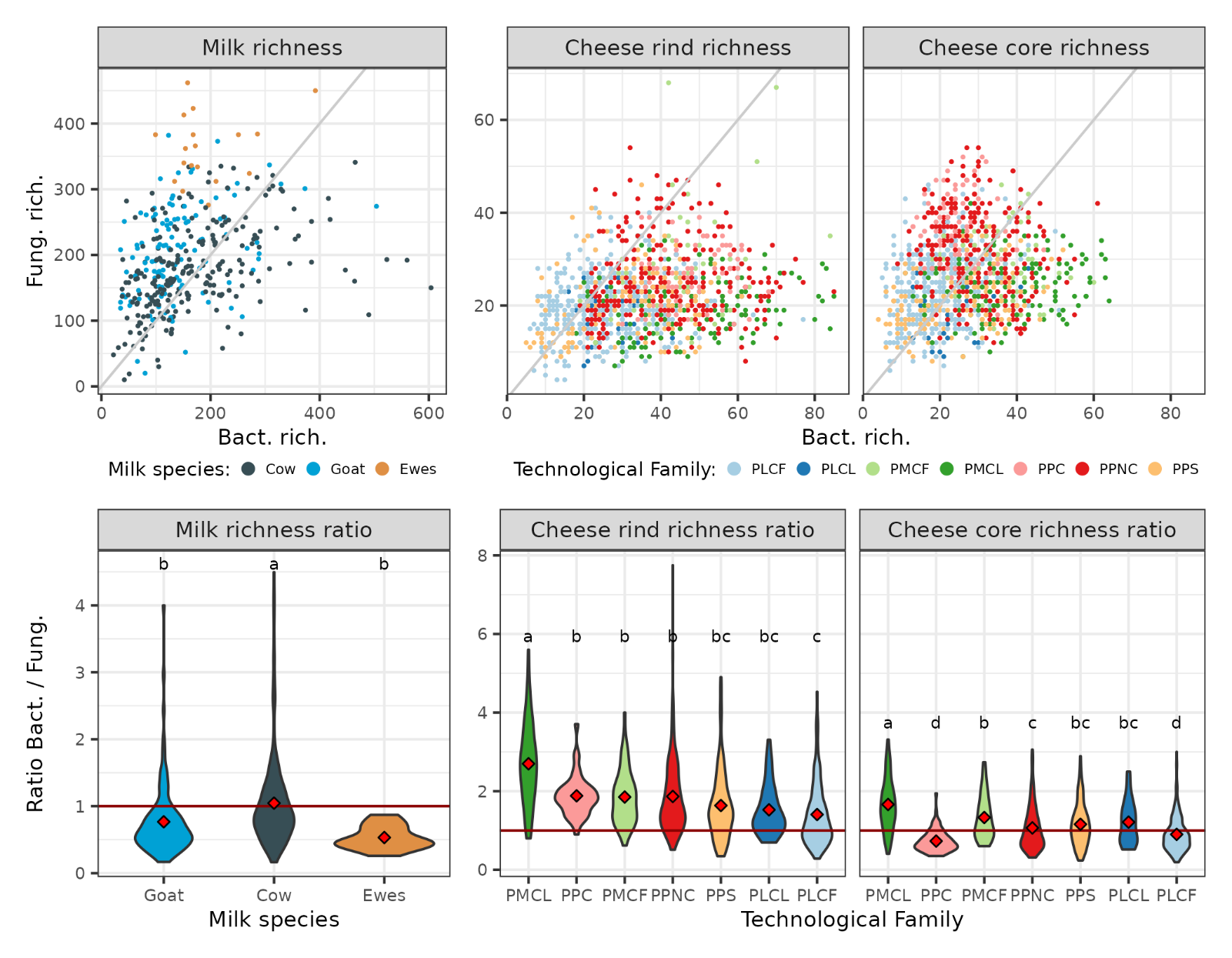


**B**

**A**

**Figure S4. α-diversity analyses based on the proportion of bacterial and fungal richness (ASV) in milk microbiota (n=370), in cheese rind microbiota (n=1,148), and in cheese core microbiota (n=1,148)**

A) Relationship between amplicon sequence variant (ASV) richness values of the bacterial community (16S ribosomal rRNA gene V3-V4 dataset) and of the fungal community (internal transcribed spacer (ITS2) region dataset. Each point represents a sample and is coloured by the animal species for milk samples and by the technology family for cheese samples. The diagonal line facilitates the visualization of the different milk species or the different technological families presenting an enrichment of one of the taxonomic groups over another.

B) Ratio of bacterial and fungal ASV richness for each sample of milks and cheeses (rinds and cores) according respectively to animal species and to technological family. Each point represents a sample. The boxplots represent the median (middle of the box), mean (red diamond), and first and third quartiles (top and bottom of the box). The red vertical line represents a ratio of 1 when communities present equal bacterial and fungal richness. The significance of differences between samples (ANOVA followed by posthoc Tukey ‘s HSD test) is marked by lowercase letters so that samples that share at least one letter are not significantly different, but samples that do not share a letter are significantly different.

Abbreviations: Internal blue mold = PPS; Soft bloomy rind = PMCF ; Lactic bloomy rind = PLCF; Lactic washed rind = PLCL; Soft washed rind = PMCL; Uncooked Pressed cheese/Semihard cheese = PPNC; Hard cooked cheese = PPC


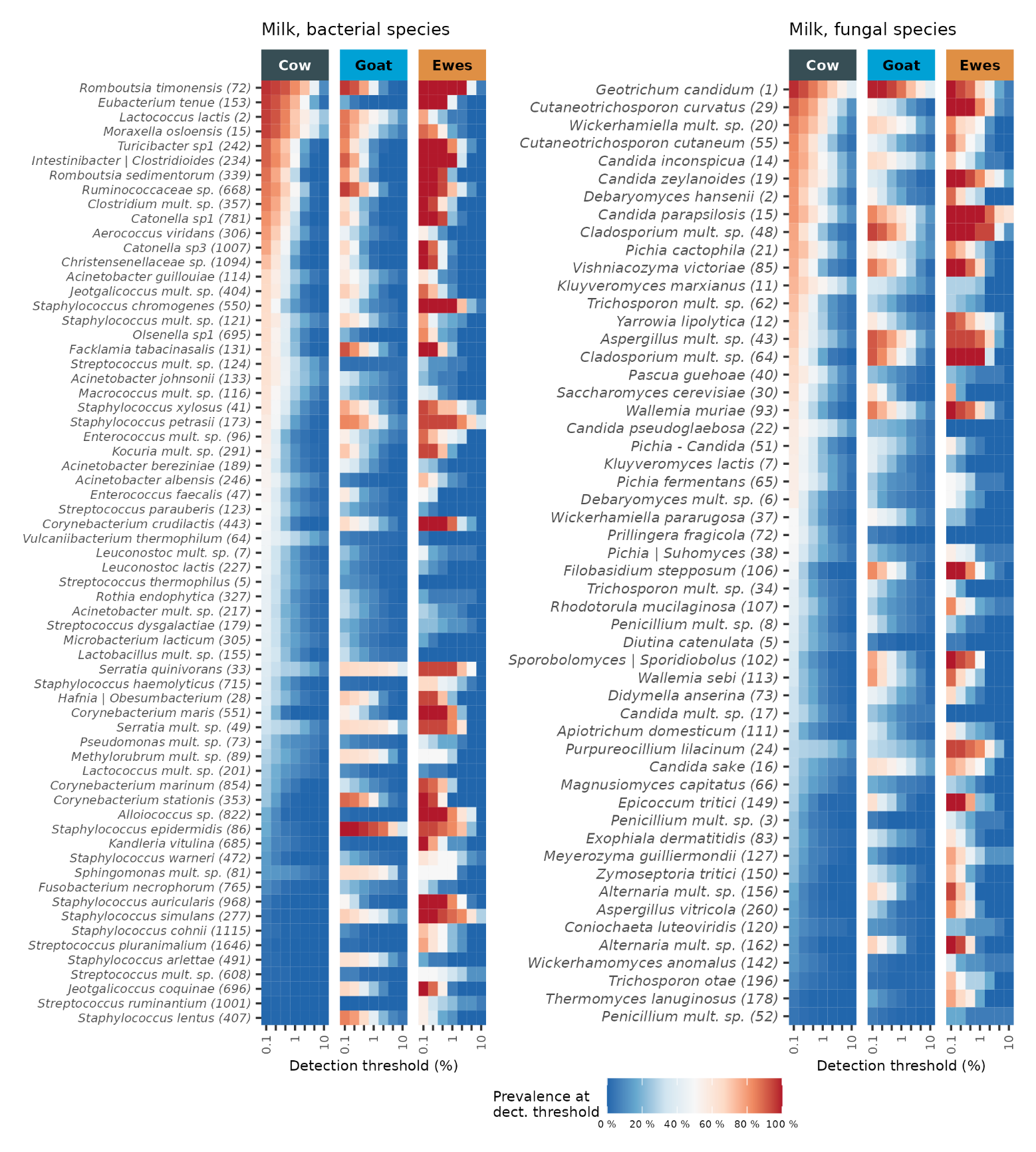
 **Figure S5A (Core microbiota of milk):** Heatmap displaying the detection threshold and prevalence of bacterial (left) and fungal (right) species across 370 milk samples clustered according to dairy animal species. Only abundant microbial species (abundance > 0.5% in at least one dairy species) appear on the heatmap (65 bacterial species, 53 fungal species). Prevalence above a given detection threshold is color-coded from dark blue (low prevalence) to dark red (high prevalence). The number between parentheses is the identifier of the most abundant ASV within the species. Taxa for which the genus is well defined, but the amplicon is shared across multiple species are identified by the suffix *mult. sp.* Taxa for which multiple genera share the same amplicon are tagged with the "|" separator (e.g., *Sporobolomyces | Sporidiobolus*). Species are sorted by decreasing prevalence (at 0.1% threshold) among cow milk samples.


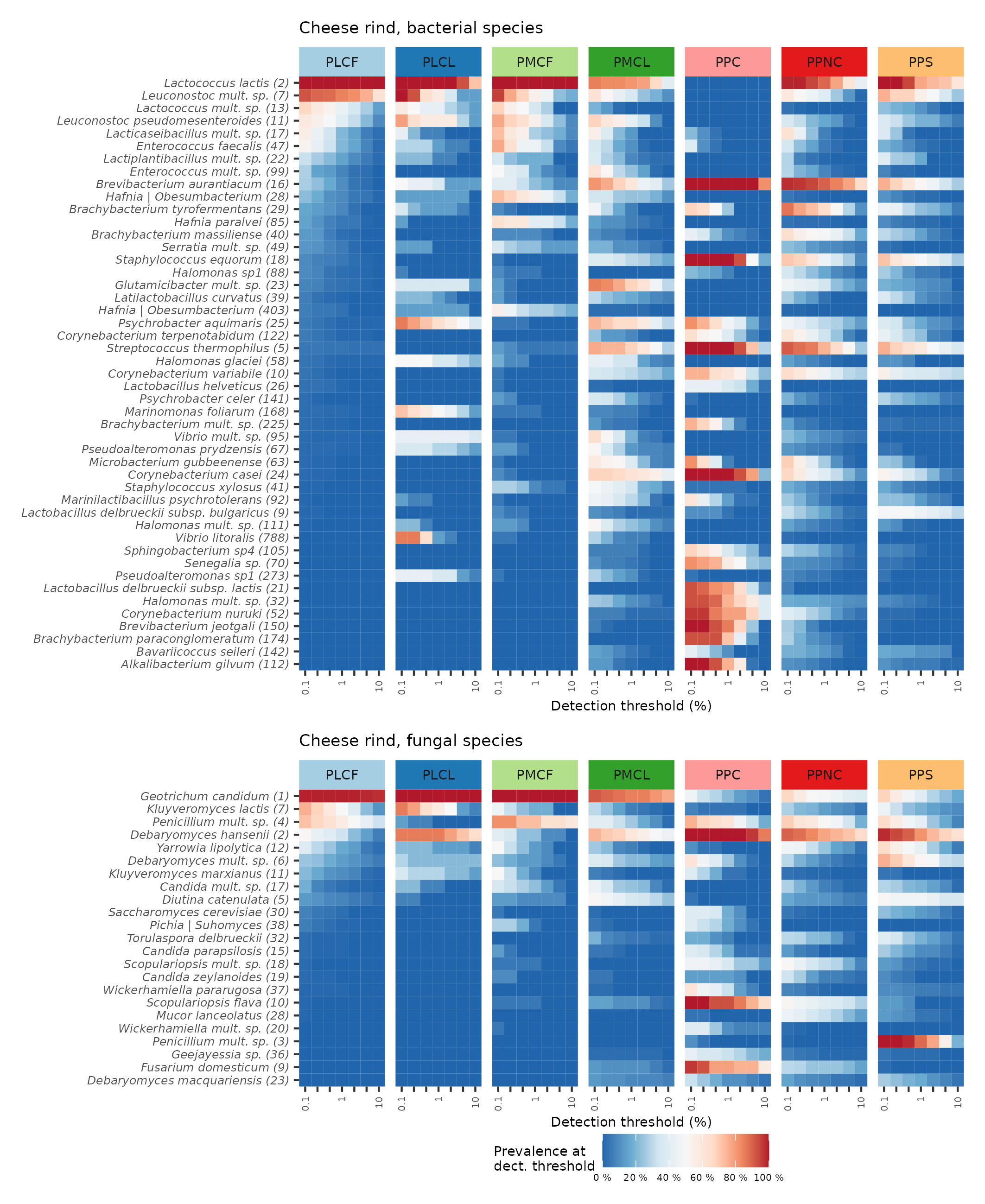


**
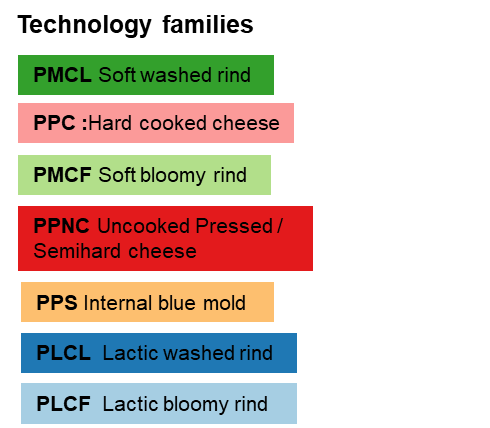
**

**Figure S5B (Core microbiota of cheese rind):** Heatmap displaying the detection threshold and prevalence of bacterial (top) and fungal (bottom) species across 1146 cheese rind samples clustered according to technological family. Only abundant species (abundance > 0.5% in at least one family) appear on the heatmap (47 bacterial species, 23 fungal species). Prevalence above a given detection threshold is color-coded from dark blue (low prevalence) to dark red (high prevalence). The number between parentheses is the identifier of the most abundant ASV within the species. Taxa for which the genus is well defined, but the amplicon is shared across multiple species are identified by the suffix *mult. sp.* Taxa for which multiple genera share the same amplicon are tagged with the "|" separator (e.g., *Sporobolomyces | Sporidiobolus*). Species are sorted by decreasing prevalence (at 0.1% threshold) among PLCF cheese rind samples.


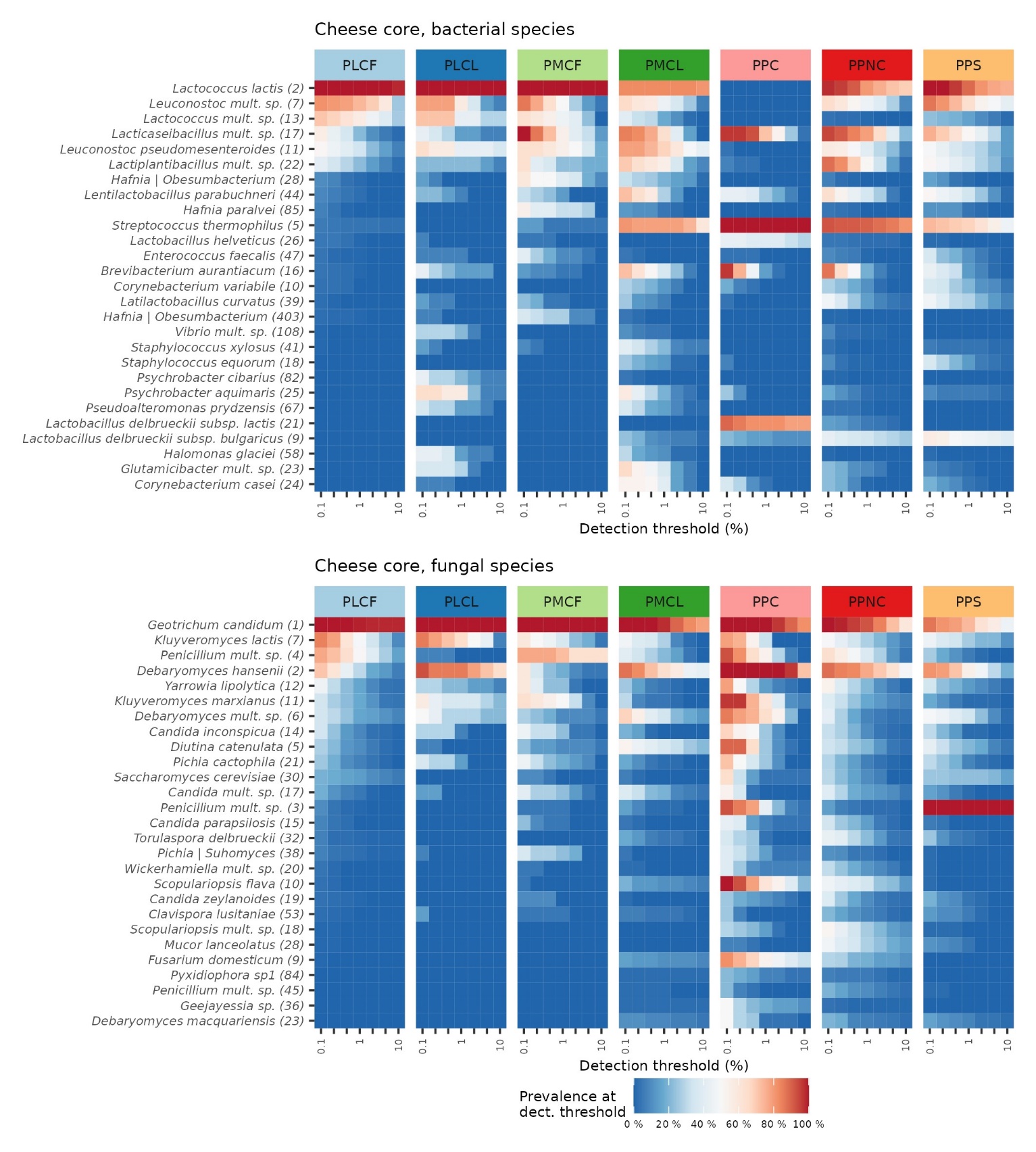


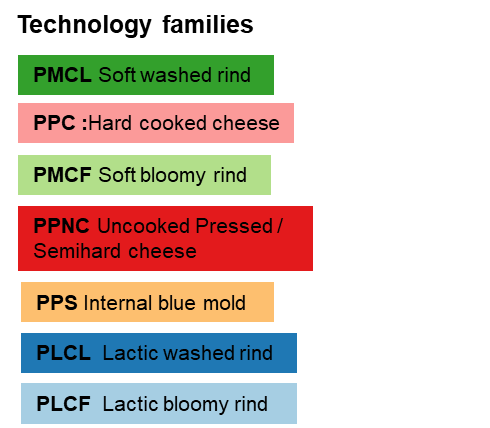


**Figure S5C (Core microbiota of cheese core):** Heatmap displaying the detection threshold and prevalence of bacterial (top) and fungal (bottom) species across 1145 cheese core samples, clustered according to technological family. Only abundant species (abundance > 0.5% in at least one family) appear on the heatmap (27 bacterial species, 27 fungal species). Prevalence above a given detection threshold is color-coded from dark blue (low prevalence) to dark red (high prevalence). The number between parentheses is the identifier of the most abundant ASV within the species. Taxa for which the genus is well defined, but the amplicon is shared across multiple species are identified by the suffix *mult. sp.* Taxa for which multiple genera share the same amplicon are tagged with the "|" separator (e.g. *Sporobolomyces | Sporidiobolus*). Species are sorted by decreasing prevalence (at 0.1% threshold) among PLCF cheese core samples.


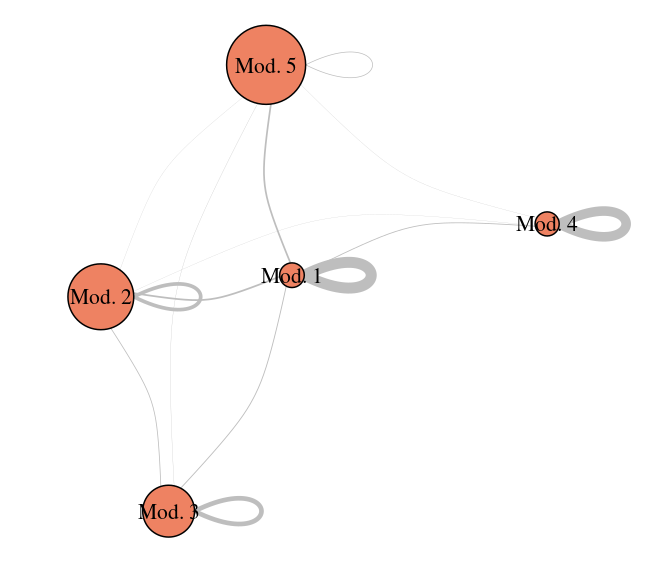

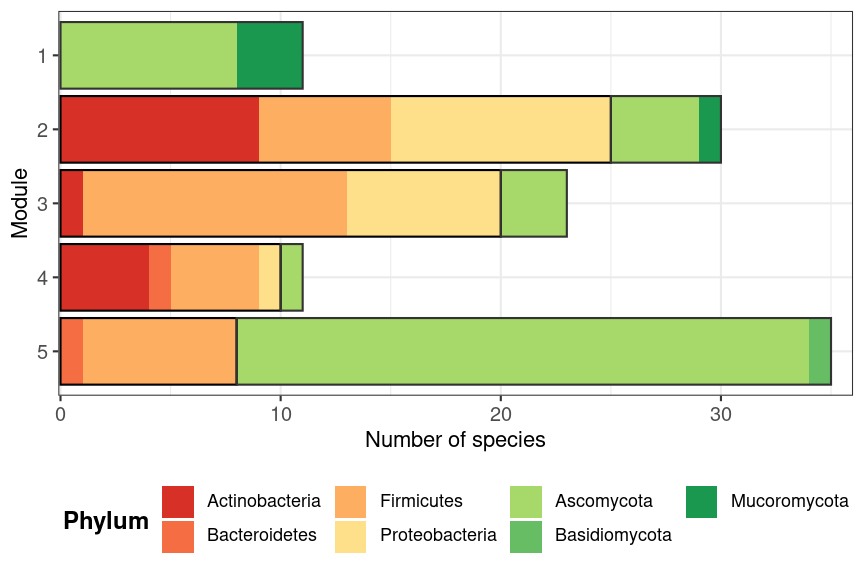


**B**

**A**

**Figure S6:** Characterization of the five modules identified in the network. A: Taxonomic composition of the modules in terms of bacterial and fungal phyla, the black rectangles correspond to Fungi and Bacteria. B: Mesoscopic summary of modules connectivity. Nodes represent the modules observed in the network, and line thickness is the mean connectivity between modules (edges for inter-module connectivity and loops for intra-module connectivity). The size of the nodes is linked to the number of species per node.

**Figure S7. Environmental, farming and technological parameters contributing to milk microbiota shaping**

A: Correlations between the amplicon sequence variant (ASV) richness of the total milk dataset (N=370), and environmental, farming and technological parameters. The parameters were sorted by category as PDO and PDO-driven variables (red dots), farming practices associated with each production (green dots) and milk sample-specific descriptors (blue dots), and then by R2 value, represented by the size of the label. Dark colours indicate significant correlations with p values below 0,05. Data for bacteria and fungi are presented in columns alternately.

B, C: Non-metric multi-dimensional scaling ordination of the milk microbial community beta-diversity (Bray-Curtis dissimilarities). Samples are coloured according to the dairy species, the effect of which is assessed through PERMANOVA analyses. c) Bacterial community (R2=0.132, p < 0.001). d) Fungal community (R2=0.060, p < 0.001)


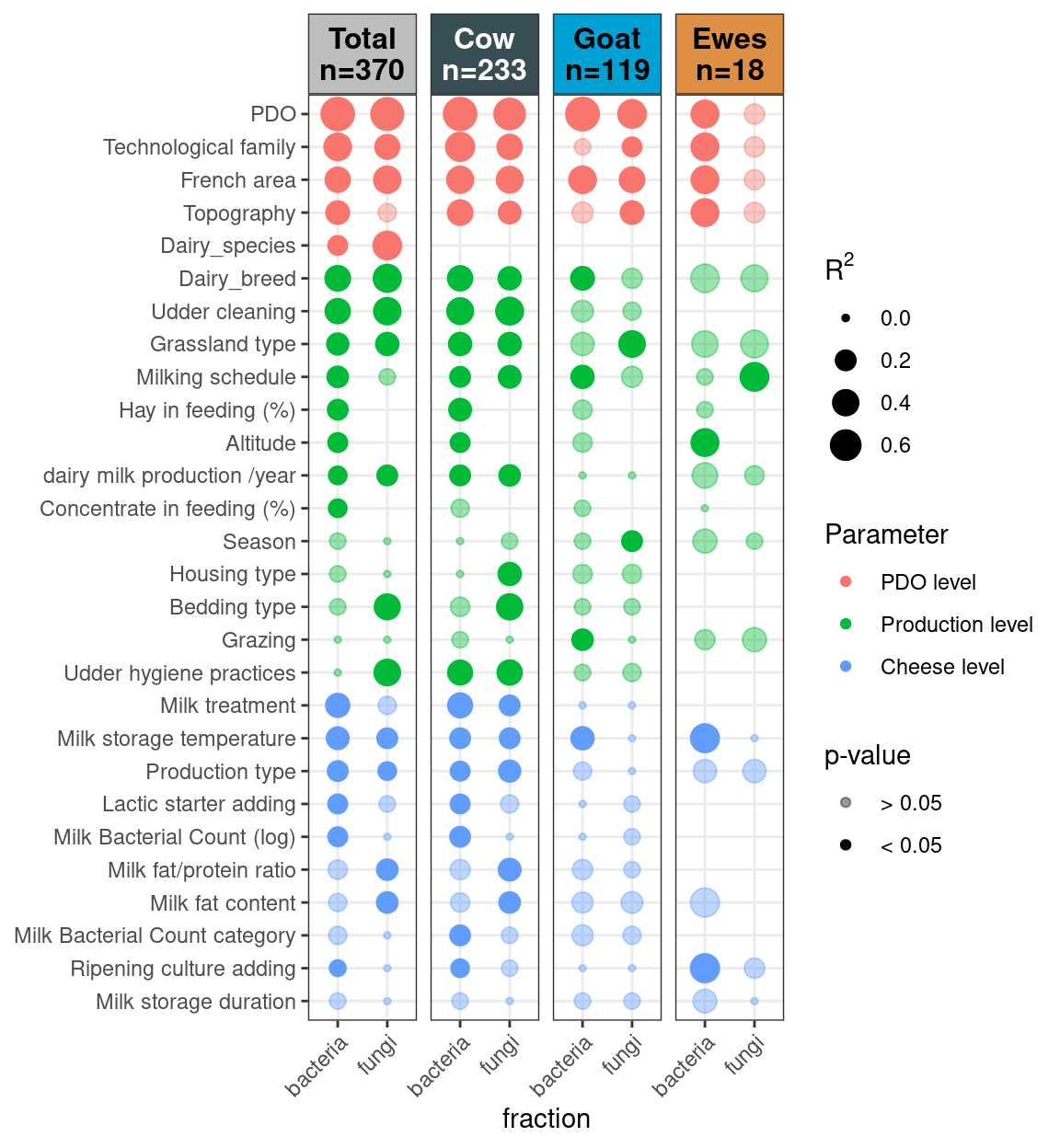


**A)**


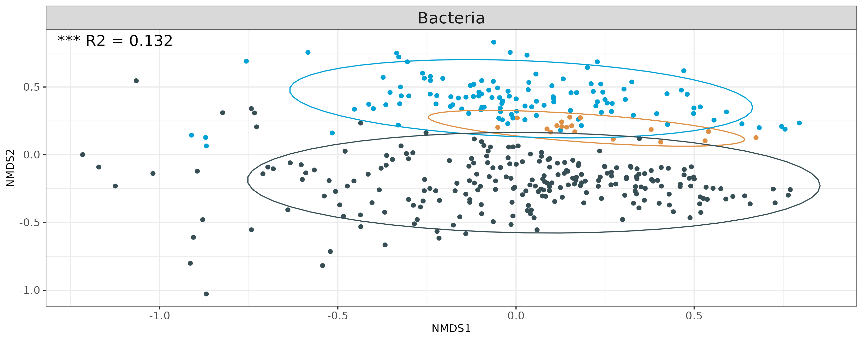

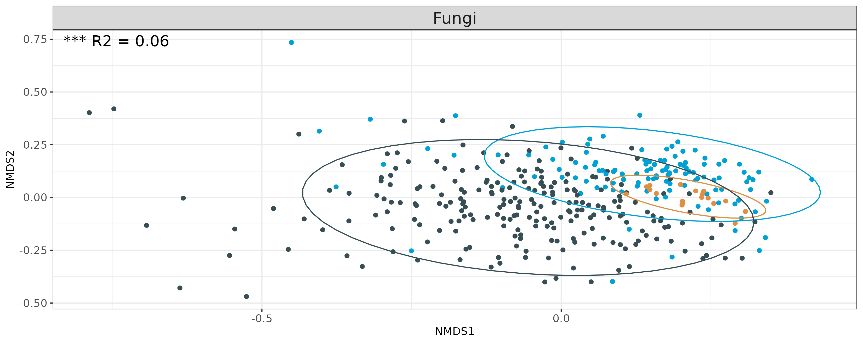


 Cow

 Goat

 Ewe

 Cow

 Goat

 Ewe

**B)**

**C)**

**Figure S8. Environmental, farming and technological parameters contributing to cheese microbiota shaping**

Correlations between the amplicon sequence variant (ASV) richness of the total cheese dataset (N=2291), and environmental, farming and technological parameters. The parameters were sorted by category as PDO and PDO-driven variables (red dots), practices associated with each production (green dots) and cheese sample-specific descriptors (blue dots), and then by R2 value, represented by the size of the label. Dark colours indicate significant correlations with p values below 0,05. Data are presented in columns alternately for bacteria and fungi.

Terminology of technological type: Internal blue mold = PPS; Soft bloomy rind = PMCF ; Lactic bloomy rind = PLCF; Lactic washed rind = PLCL; Soft washed rind = PMCL; Uncooked Pressed cheese/Semihard cheese = PPNC; Hard cooked cheese = PPC


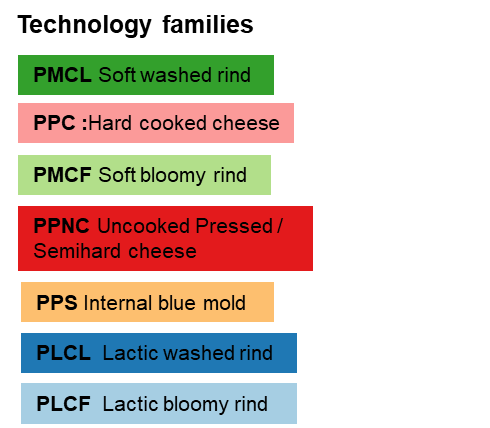

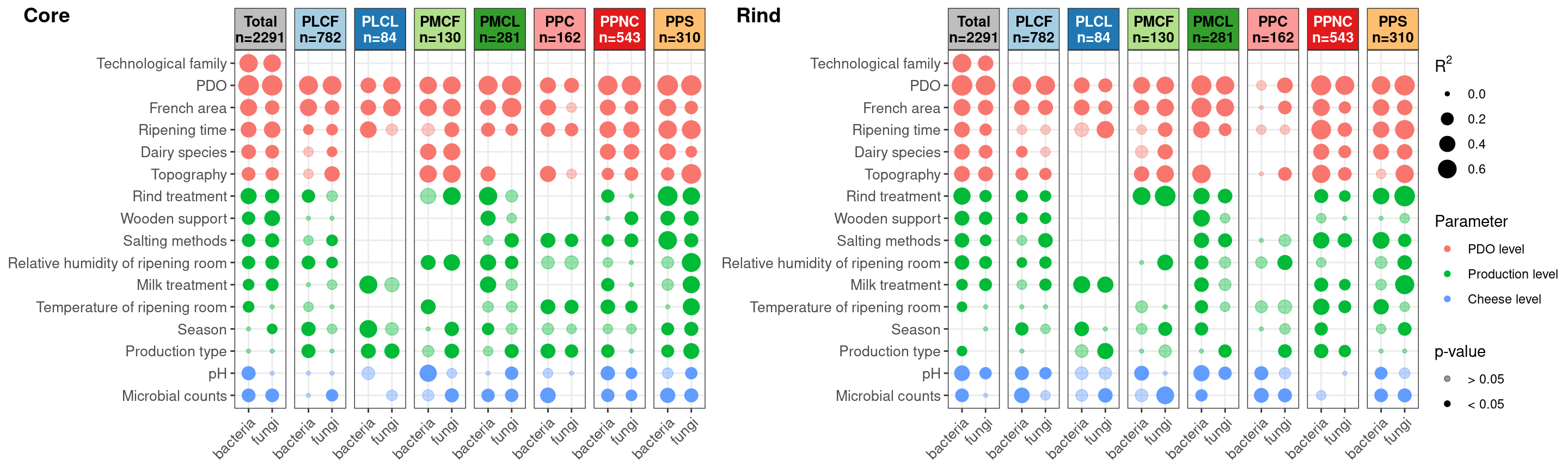
**Figure S9. Shared ASVs between milk and cheese fungal microbiota at the individual level.**


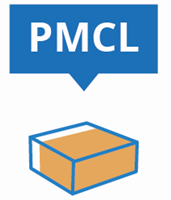

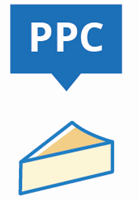

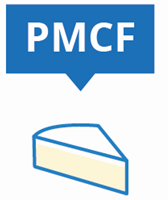

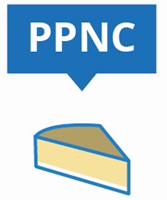

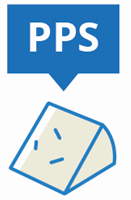

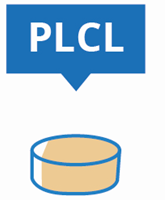

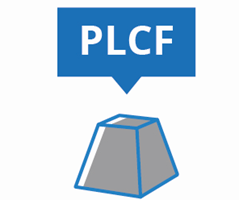


A) Contribution of milk fungal ASVs to cheese microbiota according to the cheese family. The fungal ASVs shared between a given milk sample and the derived cheese were identified i.e. a total of 740 milk-cheese core or milk-cheese surface pairs. The violin plots display the fraction of fungal ASVs shared with milk in cheese samples, for each production, according to localization (cheese rind and core) and to cheese families.

B) Fungal species assigned to the most prevalent shared ASVs in cheeses. The histograms show for each ASV the fraction of the 386 cheese productions in which this ASV was shared with the milk (yellow bar) or not shared (purple bar). The figure has been thresholded to the ASVs detected in at least 100 of the 386 cheese productions. The number in parenthesis next to the fungal species name is the number of the most abundant ASV in that species.

C) Probability of ITS_ASV19 *Candida zelanoïdes* to be shared with the cheese core according to the cheese families, the dairy species and the production type.


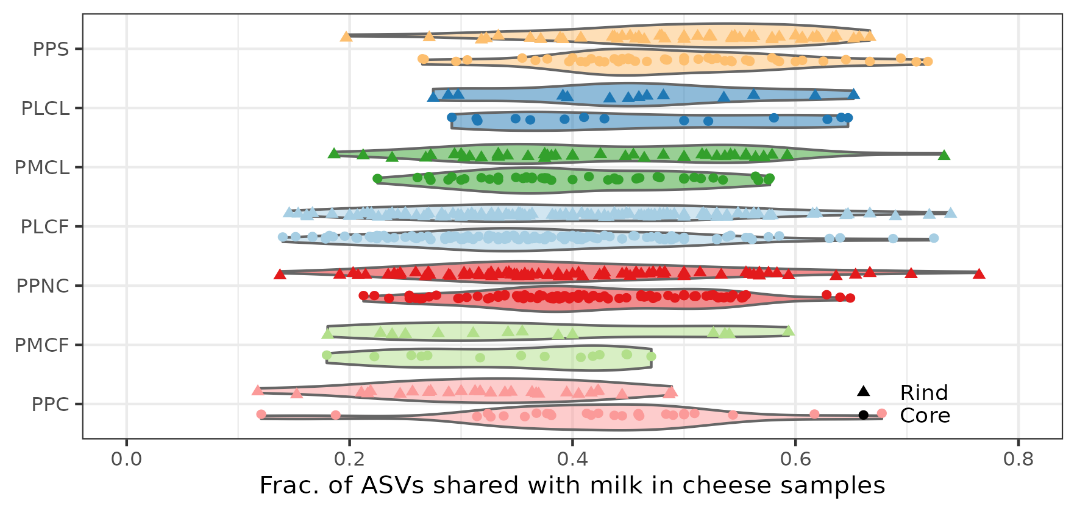
**A)**

PPS Internal blue mold

PLCL Lactic washed rind

PMCL Soft washed rind

PLCF Lactic bloomy rind

PPNC Uncooked Pressed / Semihard cheese

PMCF Soft bloomy rind

PPC Hard cooked cheese

**
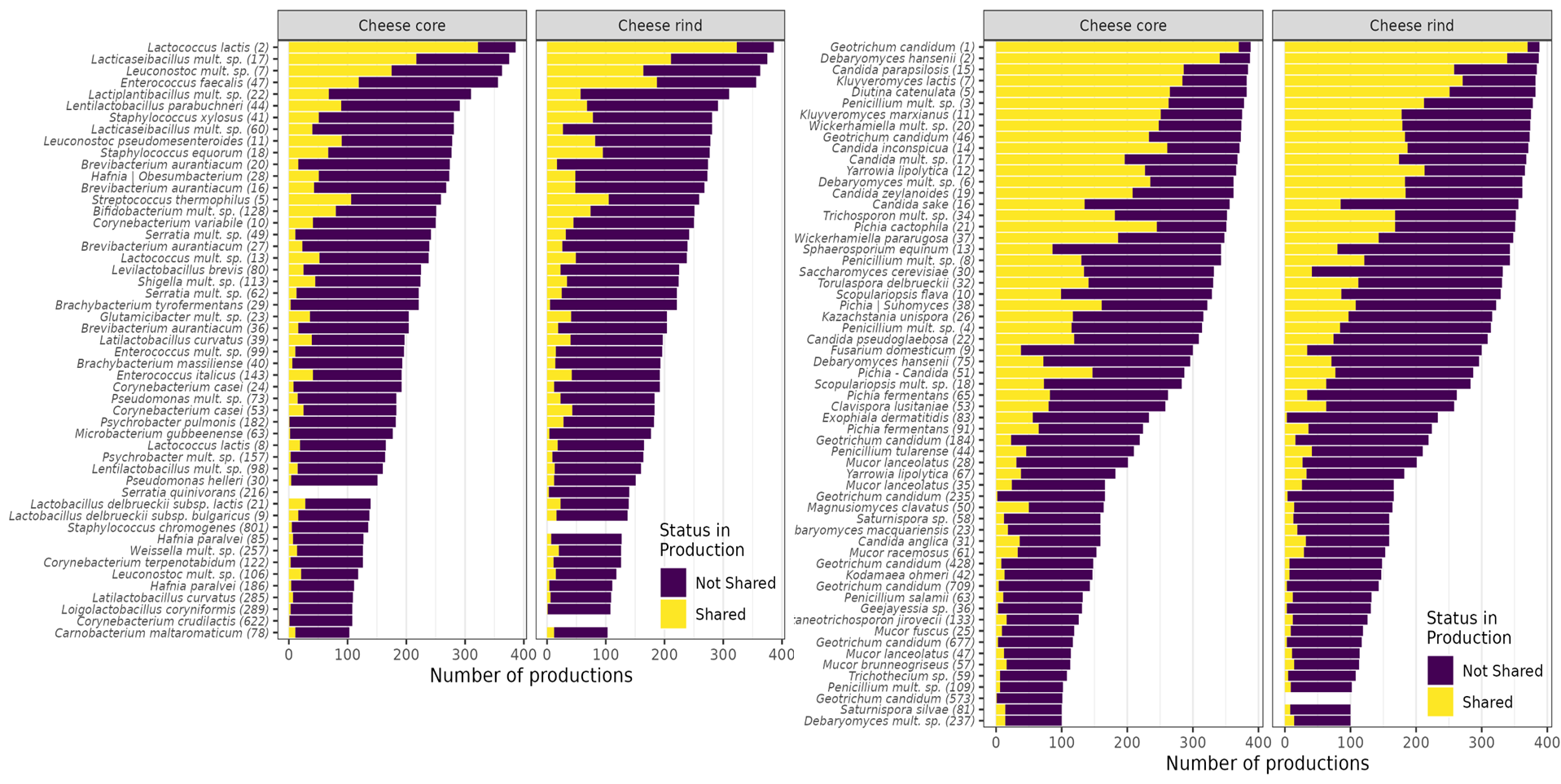
**

**B)**

ITS_ASV19 *Candida zelanoides*

**C)**

PPNC Uncooked Pressed / Semihard cheese

PPS Internal blue mold


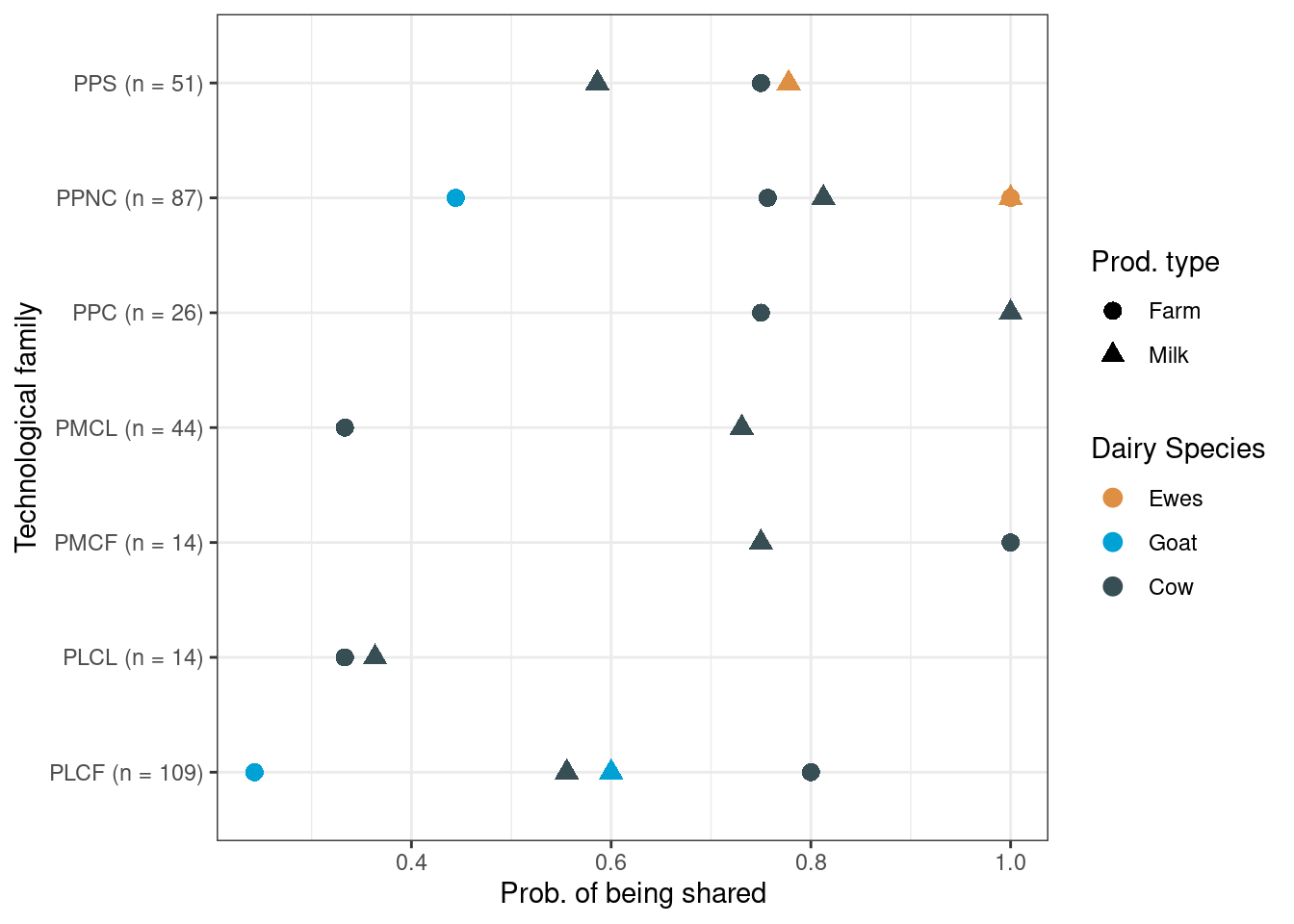


PPS_PDO39

PPNC_PDO38

PLCF Lactic bloomy rind

PLCL Lactic washed rind

PMCF Soft bloomy rind

PMCL Soft washed rind

PPC Hard cooked cheese


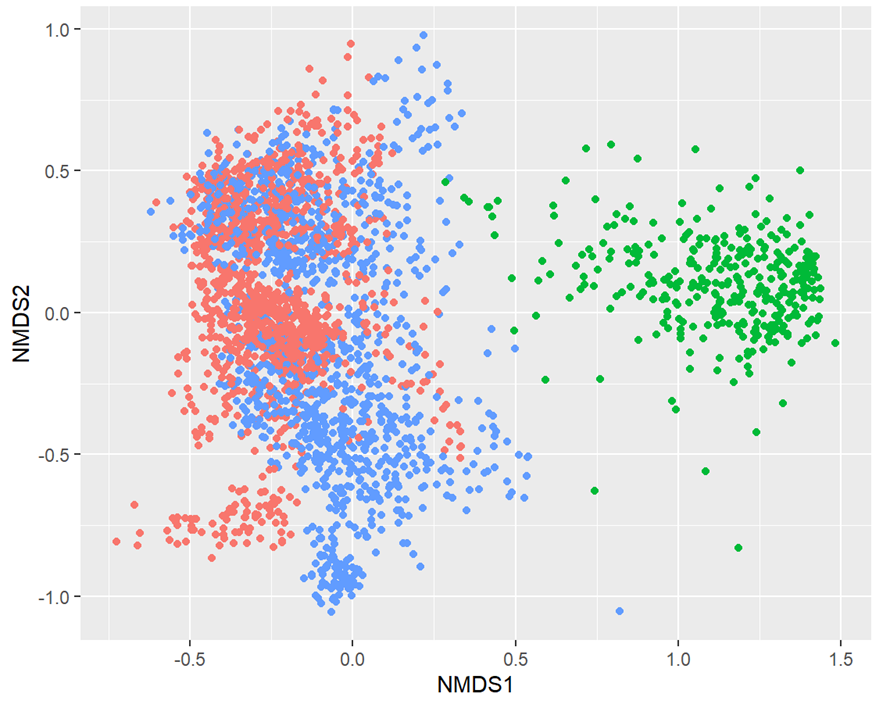
**A**  **R2=0.129*****


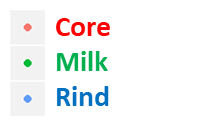


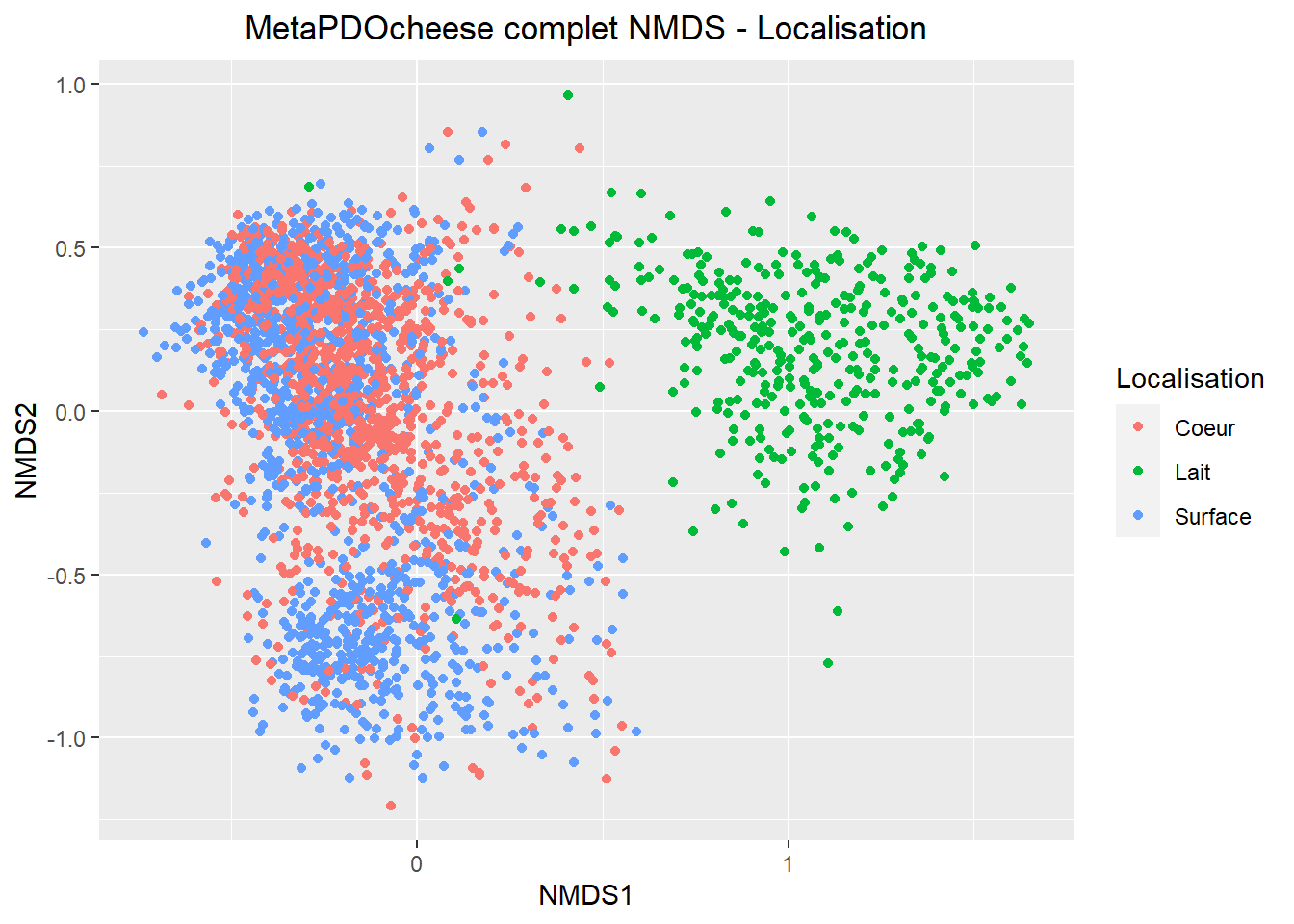


**B R2=0.088*****

**Figure S10.** Microbial community beta-diversity of the total milk and cheese dataset (N=2661) according to the type of sample (green: milk samples; blue: cheese rind samples; red: cheese core samples). Non-metric multi-dimensional scaling ordination (NMDS) based on Bray-Curtis dissimilarities. A) Bacterial community. B) Fungal community.
